# Targeted Deletion and Inversion of Tandemly Arrayed Genes in *Arabidopsis thaliana* Using Zinc Finger Nucleases

**DOI:** 10.1534/g3.113.006270

**Published:** 2013-10-01

**Authors:** Yiping Qi, Xiaohong Li, Yong Zhang, Colby G. Starker, Nicholas J. Baltes, Feng Zhang, Jeffry D. Sander, Deepak Reyon, J. Keith Joung, Daniel F. Voytas

**Affiliations:** * Department of Genetics, Cell Biology & Development and Center for Genome Engineering, University of Minnesota, Minneapolis, Minnesota 55455; † Department of Biotechnology, School of Life Sciences and Technology, University of Electronic Science and Technology of China, Chendu 610054, China; ‡ Molecular Pathology Unit, Center for Computational and Integrative Biology, and Center for Cancer Research, Massachusetts General Hospital, Charlestown, Massachusetts 02129; § Department of Pathology, Harvard Medical School, Boston, Massachusetts 02115; ** Department of Genetics, Development and Cell Biology, Interdepartmental Graduate Program in Bioinformatics & Computational Biology, Iowa State University, Ames, Iowa 50011

**Keywords:** zinc finger nuclease (ZFN), tandemly arrayed genes (TAGs), deletion, inversion, Arabidopsis

## Abstract

Tandemly arrayed genes (TAGs) or gene clusters are prevalent in higher eukaryotic genomes. For example, approximately 17% of genes are organized in tandem in the model plant *Arabidopsis thaliana*. The genetic redundancy created by TAGs presents a challenge for reverse genetics. As molecular scissors, engineered zinc finger nucleases (ZFNs) make DNA double-strand breaks in a sequence-specific manner. ZFNs thus provide a means to delete TAGs by creating two double-strand breaks in the gene cluster. Using engineered ZFNs, we successfully targeted seven genes from three TAGs on two Arabidopsis chromosomes, including the well-known *RPP4* gene cluster, which contains eight resistance (*R*) genes. The resulting gene cluster deletions ranged from a few kb to 55 kb with frequencies approximating 1% in somatic cells. We also obtained large chromosomal deletions of ~9 Mb at approximately one tenth the frequency, and gene cluster inversions and duplications also were achieved. This study demonstrates the ability to use sequence-specific nucleases in plants to make targeted chromosome rearrangements and create novel chimeric genes for reverse genetics and biotechnology.

Genome sequences of many plant species have been completed, and all contain a significant number of tandemly arrayed genes (TAGs). For example, approximately 14% of rice genes (International Rice Genome Sequencing Project 2005), 17% of Arabidopsis genes (Arabidopsis Genome Initiative 2000), and 35% of maize genes (Messing *et al.* 2004) are organized in tandem. The genetic redundancy resulting from TAGs presents a challenge for reverse genetics (Jander and Barth 2007). It is difficult, if not impossible, to eliminate TAG expression with the use of methods such as ethyl methanesulfonate mutagenesis and targeting-induced local lesions in genomes, *i.e.*, TILLING (Koornneef *et al.* 1982; McCallum *et al.* 2000), T-DNA and transposon insertional mutagenesis (Alonso *et al.* 2003; Raina *et al.* 2002; Rosso *et al.* 2003; Sessions *et al.* 2002; Woody *et al.* 2007), or RNA interference and miRNA-based gene silencing (Abbott *et al.* 2002; Alvarez *et al.* 2006; Hamilton and Baulcombe 1999; Schwab *et al.* 2006). One promising approach for studying TAGs is to make chromosomal deletions. Ionizing radiation, however, acts randomly (Li *et al.* 2001), making it difficult to recover the desired deletion. Although the Cre-Lox system has proven effective for making deletions, it relies on large LoxP T-DNA insertion populations (Zhang *et al.* 2003), which currently are unavailable for most plant species.

An alternative approach to make targeted genome deletions is to use sequence-specific nucleases. These proteins, which include zinc finger nucleases (ZFNs), transcription activator-like effector nucleases (TALENs), and meganucleases, make site-specific DNA double-strand breaks (DSBs) at a locus of interest (Christian *et al.* 2010; Kim *et al.* 1996; Smith *et al.* 2006). Repair of DSBs occurs by two pathways, namely nonhomologous end-joining (NHEJ) and homologous recombination (HR) (Puchta 2005; Puchta *et al.* 1996). NHEJ is error-prone and typically leads to insertions, deletions (indels), and substitutions at the cleavage site. In contrast, repair by HR is typically error-free because it uses a DNA template to correct the break. Of the three nuclease platforms, ZFNs have been most widely used in plants. ZFNs have been successfully used for targeted mutagenesis by NHEJ in Arabidopsis (Osakabe *et al.* 2010; Zhang *et al.* 2010) and soybean (Curtin *et al.* 2011), as well as for gene targeting by HR in tobacco (Townsend *et al.* 2009) and maize (Shukla *et al.* 2009). In addition, Petolino *et al.* (2010) reported that when a 4.3-kb beta-glucuronidase transgene was flanked by two ZFN sites, it could be efficiently deleted from the tobacco genome, thus demonstrating that ZFNs can induce chromosomal deletions of transgenes in plants. Although ZFN-mediated deletion, inversion, and duplication of endogenous chromosomal DNAs has been achieved in human cells (Lee *et al.* 2010, 2011), none of these chromosome rearrangements have yet to be demonstrated in plant cells by the use of sequence-specific nucleases.

In Arabidopsis, the receptor-like kinase (*RLK*) and the nucleotide-binding and leucine-rich repeat resistance (*R*) gene families are large and have ~600 and ~150 gene members, respectively (Meyers *et al.* 2003; Shiu *et al.* 2004). Both gene families play important roles in plant development and immunity. For example, many plant hormone receptors and almost all plant immune receptors are members of these two families. Genes in both families are organized in tandem throughout the genome. In this study, we sought to delete endogenous TAGs by using ZFNs that target three *RLK* gene clusters and one large *R* gene cluster. We successfully demonstrated targeted deletions, inversions, and duplications of multiple gene clusters as well as large chromosomal deletions exceeding 9 Mb.

## Materials and Methods

### ZFN assembly

Genomic DNA sequences of target genes were analyzed with the software ZiFiT Targeter (version 3.3) to identify ZFN sites for which ZFNs could be engineered using the Context-Dependent Assembly (CoDA) method (Curtin *et al.* 2011; Sander *et al.* 2011). DNA sequences encoding ZFNs of choice (Supporting Information, Figure S1 and Table S1) were assembled by mutagenesis and overlapping polymerase chain reaction (PCR) using standard molecular cloning procedures. For each ZFN, ZF arrays were first cloned into the yeast expression vectors pCP3 and pCP4 using available *Xba*I and *Bam*HI sites (Zhang *et al.* 2010). Then, DNA sequences for the left and right ZF arrays were excised from the yeast expression vectors with *Xba*I and *Bam*HI and moved into the pZHY013 entry clone using the *Xba*I-*Bam*HI and *Nhe*I-*Bgl*II sites, respectively (Figure S2). pZHY013 contains an obligate *Fok*I heterodimer architecture (Miller *et al.* 2007), and the ZFNs are linked by a T2A translational skipping sequence. The plant ZFN expression vectors were made using a Gateway LR reaction between the aforementioned entry clones and the pFZ19 destination vector (Zhang *et al.* 2010).

### Transgenic plants and expression of ZFNs

*Agrobacterium tumefaciens* GV3101/pMP90 was transformed with pFZ19 plasmids containing the ZFNs. The transformed *A. tumefaciens* strain was then used to transform Arabidopsis Col-0 (wild-type) plants using the floral dip method (Clough and Bent 1998). T1 transgenic plants were selected by growing the sterilized seeds on 0.5× MS solid medium (0.8% agar) that contained 100 μg/mL timentin (PlantMedia) and 20 μg/mL hygromycin B (Roche). For inducing ZFN expression, 20 µM β-estradiol (Sigma-Aldrich) was included in the medium.

### ZFN activity measurement

One-week-old seedlings grown on MS medium with estradiol were harvested for DNA extraction with the CTAB DNA isolation method (Stewart and Via 1993). Eight T2 transgenic plants from the same T1 parent were bulked to represent each sample, whereas eight wild-type plants were bulked as the negative control.

To detect ZFN activity, an enrichment PCR procedure was used. To summarize, ~500 ng of genomic DNA from each sample was digested overnight (16 hr) with 1 µL of *Dde*I (for At1g53-ZFN), *Afl*II (for At1g70-ZFN), *Bfa*I (for At4g16-ZFN), *Bmg*BI (for At3g21-ZFN), or *Ear*I (for At5g01-ZFN) in a 20-µL reaction volume. Four microliters of digested genomic DNA was used for PCR amplification of the corresponding ZFN target sites in a 25-µL reaction volume. Ten microliters of unpurified PCR product was then digested with 1 µL of the same restriction enzyme in a 40-µL reaction volume for 12−16 hr. Digested products were resolved by electrophoresis in 1.5% agarose gels; mutations created by ZFNs were evidenced as undigested PCR products.

An alternative method to detect and measure ZFN activity by restriction digestion is similar to enrichment PCR except that the genomic DNA digestion step is omitted. Four microliters of 50 ng/µL genomic DNA was directly used for PCR and subsequent digestion by the corresponding restriction enzyme. The frequency of ZFN-mediated mutagenesis was measured by quantifying the percentage of undigested PCR product that was resolved by electrophoresis on a 1.5% agarose gel.

ZFN activity also was measured by the Surveyor assay in which T7 endonuclease was substituted for Cel-I (Guschin *et al.* 2010). In summary, PCR products amplified from genomic DNA templates were purified with a QIAquick PCR purification kit. For each sample, ~500 ng of purified PCR product was mixed with NEB buffer 2 in a 30-µL volume. To promote heteroduplex formation, PCR amplicons were denatured and reannealed using the following regime: 95° for 5 min, 95° to 85° at −1.5°/sec, and 85° to 25° at −0.1°/sec. One microliter of T7 endonuclease (NEB) was added to each sample for digestion at 37° for 1 hr. The digested products were resolved by electrophoresis in 1.5% agarose gels, and the frequency of ZFN-mediated mutagenesis was quantified as described previously (Guschin *et al.* 2010).

### Sequence confirmation of mutagenesis and deletions

Both undigested PCR products (for assessing mutagenesis) and PCR products (for detecting deletions, inversions, and duplications) were purified with the QIAquick Gel Extraction Kit. The purified DNA products were then cloned using either the pCR8/GW/TOPO TA Cloning Kit or the pCR2.1 Original TA Cloning Kit (Invitrogen). Multiple clones for each experiment were randomly picked and subjected to DNA sequencing.

### Deletion frequency measurements

A method similar to digital PCR analysis (Lee *et al.* 2010) was used to estimate deletion frequencies. Genomic DNA samples were serially diluted (in a 3× gradient) in distilled water, and they were used for PCR via the use of deletion-specific primer pairs (Table S2). The same DNA samples were used to amplify a fragment of the *ADH1* gene as a genomic DNA copy number control. In each case, the difference of dilution factors for both deletion PCR and control PCR was used to calculate deletion frequency.

## Results

### Strategy for targeted deletion of TAGs

**Figure 1:**
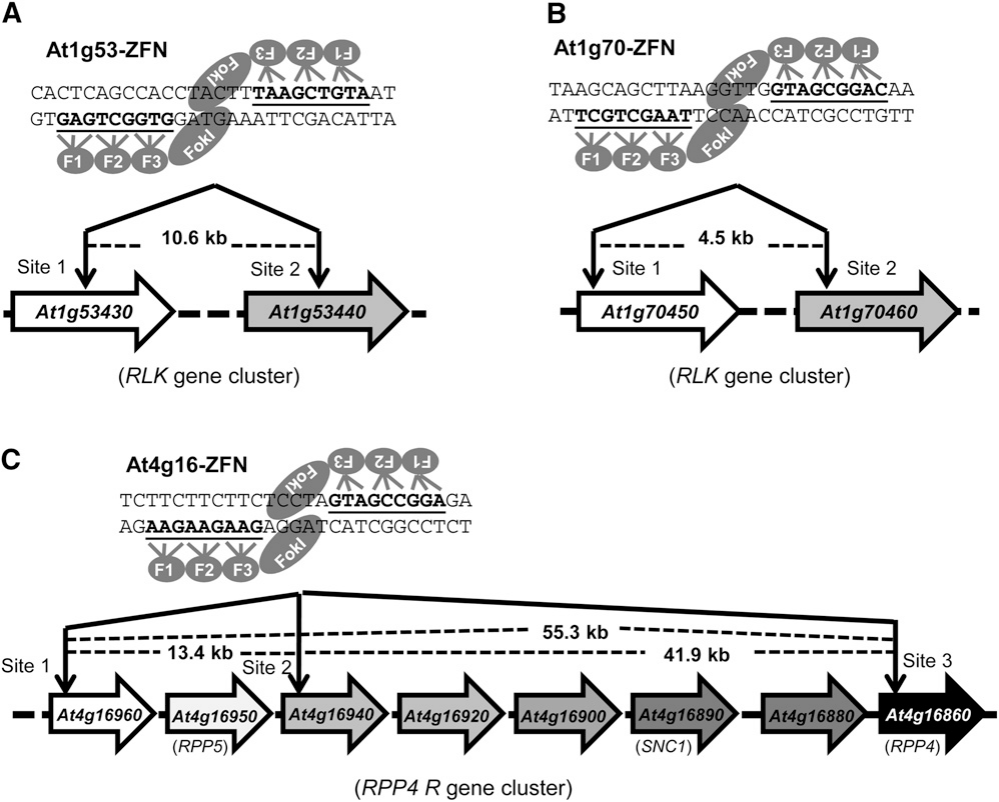
Schematic of target genes and ZFN sites. (A) The At1g53-ZFN targets both *At1g53430* and *At1g53440* in the 14th exon of each gene. (B) The At1g70-ZFN targets *At1g70450* in the 1st exon and *At70460* in the 2nd exon. (C) The At4g16-ZFN targets three sites in the *RPP4* R gene cluster: the 1st exon of *At4g16960*, the 5′ UTR of *At4g16940*, and the 1st exon of *At4g16860*. Illustration of ZFN pairs depicts the DNA recognition triplets for each zinc finger. The zinc finger binding sequences are underlined, and the distance between cleavage sites is shown.

Chromosomal deletions can be stimulated by two coordinated DSBs (Petolino *et al.* 2010). In our strategy, we used a single pair of ZFNs, which because of the high sequence similarity among TAGs, created two or more DSBs. Five ZFNs were engineered using the CoDA method to target three *RLK* gene clusters, the *RPP4 R* gene cluster, and the *ASK8* gene cluster (Figure 1 and Figure S3). These five ZFNs were expected to induce a total of 13 DNA DSBs in targeted exons of the Arabidopsis genome. The resulting size of predicted deletions ranged from a few to more than 50 kb. In addition to inducing deletions, ligation of broken chromosomes can create novel gene fusions. However, because NHEJ is error-prone, we anticipated that indels would be introduced at the cleavage site, thus rendering some of the deleted TAGs nonfunctional.

### Detection of ZFN-induced mutagenesis

For all ZFNs used in this study, both the left and right ZFNs were expressed from an estradiol-inducible promoter and separated by a “self-cleaving” T2A peptide, which promotes production of two proteins from one mRNA by a translational skipping mechanism (Szymczak *et al.* 2004). T1 transgenic plants were screened on MS medium containing both hygromycin (to identify transgenic plants) and β-estradiol, which allowed for induction of ZFN transgenes. Detection of ZFN-induced mutations was performed by the use of enrichment PCR (Qi *et al.* 2013). Indels introduced by ZFNs frequently occur in the “spacer” region, where the *Fok*I nuclease domains dimerize and cleave the DNA. Thus, if there is a unique restriction enzyme site in the spacer, this restriction enzyme site will likely be destroyed through ZFN-mediated mutagenesis and thus render the DNA uncuttable by the restriction enzyme.

For each locus evaluated, T1 plants in which ZFNs were induced by estradiol were pooled together and genomic DNA was extracted. For the At1g53-ZFN, which targets the *At1g53430-At1g53440* gene cluster, ZFN activity was detected at the *At1g53430* target site (Figure S4A). Similarly, the At1g70-ZFN and At4g16-ZFN were found to be active at the *At1g70450-At1g70460* gene cluster and the *RPP4* gene cluster, respectively (Figure S4, B and C). We did not detect ZFN activity for the At3g21-ZFN and the At5g01-ZFN (Figure S3, data not shown). These data suggest that three of the five ZFN pairs are functional, and their activity can be temporally controlled by the estradiol-inducible promoter (Figure 1). We thus focused on the three active ZFNs for further study.

### Quantification of ZFN activity at seven endogenous loci

**Figure 2:**
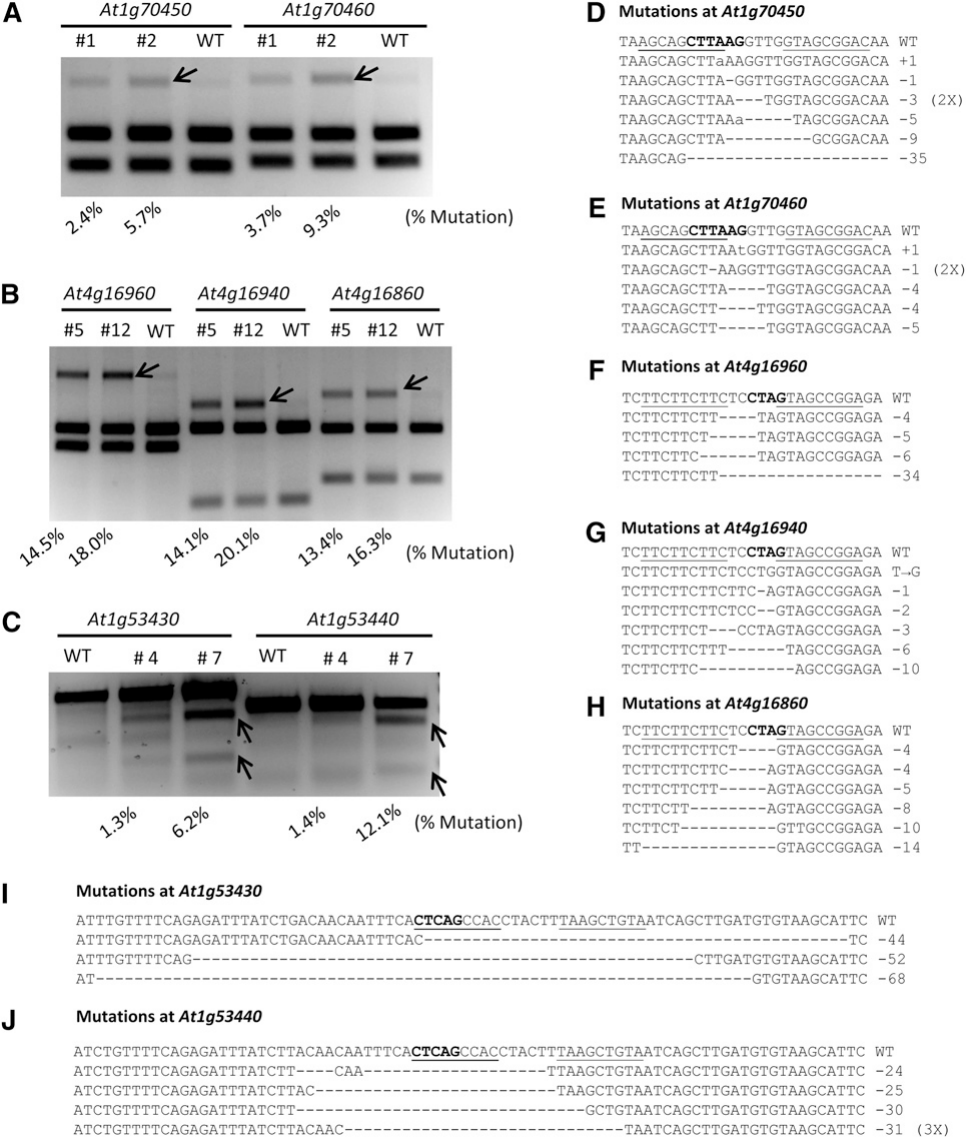
ZFN activity at seven endogenous loci. (A) At1g70-ZFN activity at target sites in *At1g70450* and *At1g70460*. PCR products were digested with *Afl*II. (B) At4g16-ZFN activity at target sites in *At4g16960*, *At4g16940*, and *At4g16860*. PCR products were digested with *Bfa*I. (C) At1g53-ZFN activity at target sites in *At1g53430* and *At1g53440* as measured by the T7 endonuclease assay. For both restriction digestion assays (A and B) and the T7 endonuclease assay (C), mutagenesis frequencies (shown at the bottom of the figures) were determined by measuring the signal intensity of each band using the Labworks analysis software. (D-J) ZFN-induced mutations at seven endogenous target sites with different mutation types indicated. The restriction enzyme sites used for activity measurement are marked in bold letters. The uncut PCR products (from A and B, and Figure S2) were cloned and sequenced to reveal ZFN-induced mutations at each target site.

We screened multiple T2 populations of Arabidopsis plants transformed with each ZFN pair and selected two independent lines showing high ZFN activity. These transgenic plants were used to quantify ZFN activity based on the level of NHEJ mutagenesis in whole seedlings. Note that the frequency of mutation observed by enrichment PCR is influenced by the position of the restriction enzyme site. The *Afl*II and *Bfa*I sites are very close to the middle of the spacer in the At1g70-ZFN and At4g16-ZFN recognition sites, respectively. In these cases, we reasoned the percentage of uncut DNA by these restriction enzymes should approximate the ZFN-mediated mutagenesis frequency. On the other hand, the *Dde*I site is farther away from the spacer in the At1g53-ZFN target site. Because only a fraction of mutagenesis events are likely assessed by measuring loss of the *Dde*I site, the T7 endonuclease assay was instead used to measure activity of the At1g53-ZFN. Activity of the three ZFNs at seven endogenous loci ranged from a few percent to 20% (Figure 2, A−C), with the At4g16-ZFN having the greatest activity (13.4–20.1%). Differences observed in ZFN activity among different transgenic plants is likely due to different expression levels of the ZFN transgenes, depending on the chromosomal site of T-DNA integration.

To further confirm activity of each ZFN, DNA fragments resistant to restriction enzyme digestion were cloned and sequenced (Figure 2, A and B). The sequencing results revealed ZFN-induced mutations at all seven target loci, with small deletions (1−10 bp) being the most prevalent (Figure 2, D−J). Because the *Dde*I restriction site is farther away from the spacer, deletions recovered at *At1g53430* and *At1g53440* were typically larger (24−68 bp; Figure 2, I and J and Figure S5).

### Deletion of TAGs by ZFNs

**Figure 3:**
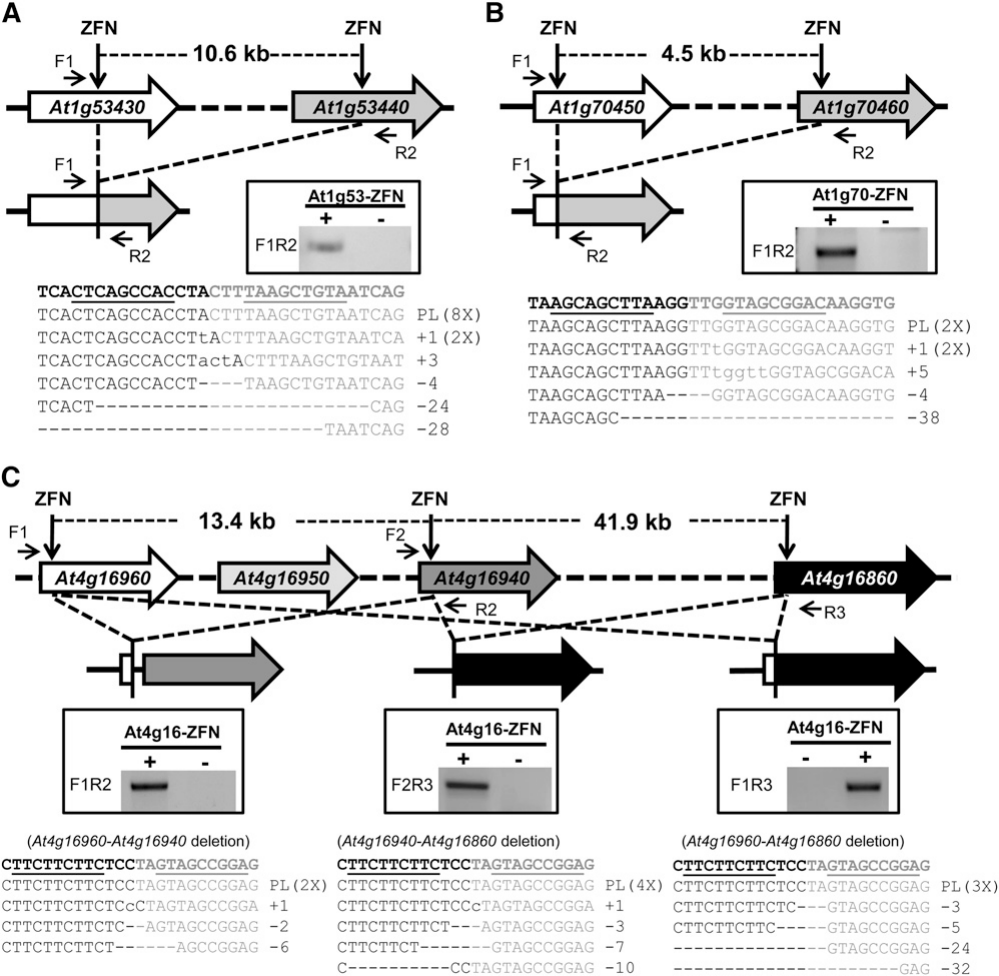
Deletion of gene clusters by ZFNs. (A) Deletion of the *At1g53430-At1g53440* cluster by the At1g53-ZFN. (B) Deletion of the *At1g70450-At1g70460* cluster by the At1g70-ZFN. (C) Three types of deletions at the *At4g16960-At4g16860* gene cluster generated by the At4g16-ZFN. The gene clusters and resulting deletions are depicted. Deletion events were confirmed by PCR, as shown in windows in the middle of each panel. The positions of PCR primers are indicated by arrows. PCR products were subsequently cloned and sequenced. The sequencing results shown in the lower panels confirmed perfect ligations after loss of the intervening DNA or ligations with mutations at the ZFN cleavage site.

The observed mutations at all seven target sites indicate that ZFN-induced DSBs were generated. To examine deletion of TAGs as an outcome to ZFN activity, we conducted PCR using specific primers flanking all ZFN target sites, such that the production of PCR products would indicate the presence of deletions (Figure 3, A−C). With this strategy, we detected deletions of the *At1g53430* gene cluster (Figure 3A) and the *At1g70450* gene cluster (Figure 3B), as well as three different types of deletions at the *RPP4* gene cluster (Figure 3C). We further confirmed these deletions by cloning the deletion-specific PCR products and sequencing randomly selected clones (Figure 3, A−C, lower panels). Not only did we observe indels at the site of deletions, we also observed perfect ligations of two sticky ends derived from ZFN-cleaved DNA (Figure 3, A−C). These events are most likely due to the presence of compatible overhangs. Taken together, these data suggest that deletions of up to 55 kb can be made by ZFNs on different Arabidopsis chromosomes.

### Large chromosomal deletions by ZFNs

**Figure 4:**
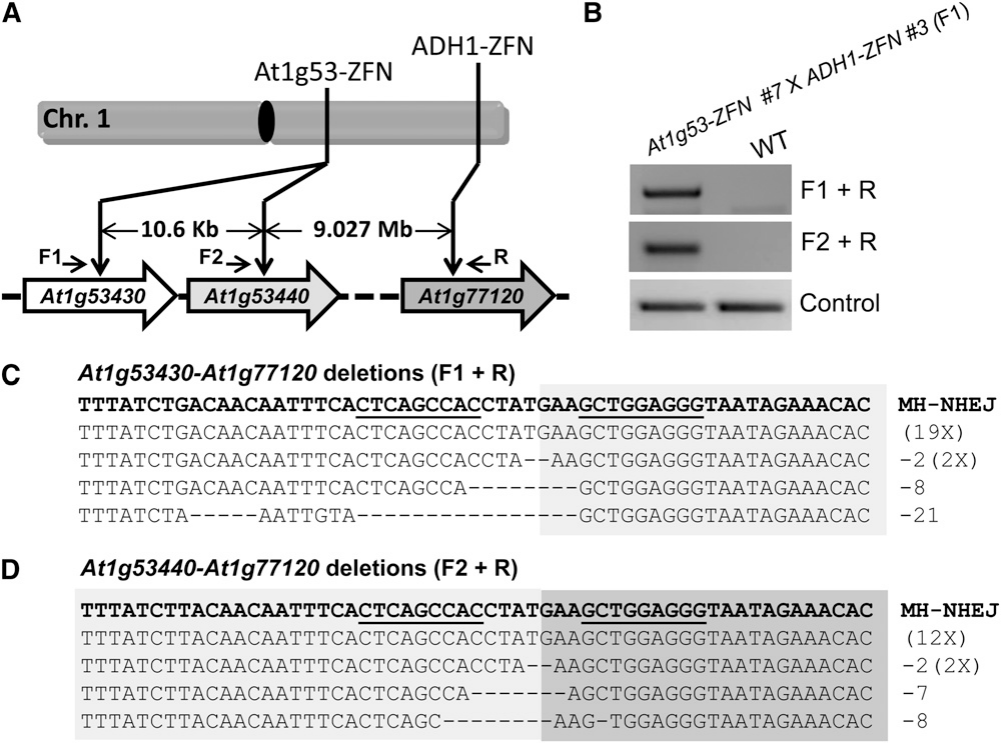
Large chromosomal deletions by ZFNs. (A) Schematic of ZFN targets on the right arm of Arabidopsis chromosome 1. The distance between the ZFN sites is shown and the positions of primers used to confirm large deletions are indicated. (B) PCR confirmation of large chromosomal deletions. The F1 and R primers amplify the junction fragment of the deletion of 9.037 Mb; primers F2 and R amplify the junction fragment of the deletion of 9.027 Mb (upper panels). PCR amplification of a part of the *ADH1* gene was used as a genomic DNA control (lower panel). F1 seedlings generated from the cross between At1g53-ZFN #7 line and ADH1ZFN #3 line were treated with estradiol, and the wild-type plants served as a negative control. (C) Sequenced clones indicative of large chromosomal deletions between *At1g53430* and *At1g77120*. (D) Sequenced clones indicative of large chromosomal deletions between *At1g53440* and *At1g77120*. The DNA sequences resulting from perfect ligation of DNA ends are shown in the first line of the text boxes; deletions with indels are shown below. ZFN binding sequences are underlined. MH-NHEJ, end-joining that appears to have been facilitated by microhomology.

Because we achieved deletions of TAGs spanning up to 55 kb, we next tested whether much larger chromosomal deletions could be generated. To do so, we took advantage of an existing ZFN (ADH1-ZFN), which we previously engineered to target the *ADH1* gene (*At1g77120*) at the end of chromosome 1 (Zhang *et al.* 2010) (Figure 4A and Figure S6A). Simultaneous expression of At1g53-ZFN and ADH1-ZFN could potentially result in chromosomal deletions as large as 9 Mb, almost one-third the length of Arabidopsis chromosome 1 (Figure 4A). We first screened independent estradiol-inducible *ADH1-ZFN* lines and identified the *ADH1-ZFN* #3 line as showing strong estradiol-inducible mutagenesis activity (Figure S6B). We also mapped the *ADH-ZFN* transgene insertion site in this line to chromosome 2 to aid in genotyping (Figure S6C). We then obtained a homozygous T3 *ADH1-ZFN* #3 line for use in our experiments.

To test for chromosomal deletions, we crossed the homozygous *At1g53-ZFN* #7 T2 line and the *ADH1-ZFN* #3 T3 line. F1 plants were obtained, and both ZFNs were induced by growing them on estradiol-containing MS medium. As with the gene cluster deletions, PCR was used to detect the large chromosomal deletions, and deletions were detected for both *At1g53430-At1g77120* and *At1g53440-At1g77120* (Figure 4B). Resulting PCR products were cloned and sequenced (Figure 4, C and D). Interestingly, the most prevalent product was a ligation of a 7-bp spacer sequence. We predict this product resulted from microhomology-based NHEJ using only 1 bp of microhomology (Figure S7). The resulting 7-bp spacer would be expected to be cut inefficiently by the hybrid ZFN, which contains At1g53-ZFN-left and ADH1-ZFN-right monomers (Figure S7). This may explain the PCR product’s predominance.

### Frequency of ZFN-induced chromosomal deletions

Having demonstrated ZFN-induced chromosomal deletions ranging from a few kilobases to 9 Mb, we next sought to estimate their frequency of occurrence. We adapted a digital PCR method used to measure deletion frequencies in human cells (Lee *et al.* 2010). In our case, deletion frequency was detected by PCR using a series of genomic DNA dilutions as templates. Amplification of *ADH1* was used as an internal control for DNA copy number. As summarized in Table 1, the frequency of gene cluster deletions was approximately 1%—positively correlated with ZFN activity and negatively correlated with the length of the deletions. As the length of deletion increased to 9 Mb, the frequency decreased to <0.1%.

**Table 1:** Frequency of ZFN-induced chromosomal deletions

Transgenic Lines	*At1g53430* to *At1g53440* (deletion of 10.6 kb)	*At1g70450* to *At1g70460* (deletion of 4.5 kb)	*At4g16940* to *At4g16860* (deletion of 41.9 kb)	*At4g16960* to *At4g16860* (deletion of 55.3 kb)	*At1g53440* to *At1g77120* (deletion of 9.027 Mb)	*At1g53430* to *At1g77120* (deletion of 9.037 Mb)
*A1g53-ZFN #4*	0.3%					
*At1g53-ZFN #7*	1%					
*At1g70-ZFN #1*		3%				
*At1g70-ZFN #2*		3%				
*At4g16-ZFN #5*			1%	1%		
*At4g16-ZFN #12*			3%	3%		
*ADH1-ZFN #3 X*					0.046%	0.137%
*At1g53-ZFN #7**^a^*						

ZFN, zinc finger nucleases.

a F1 plants.

### Targeted inversions of gene clusters

**Figure 5:**
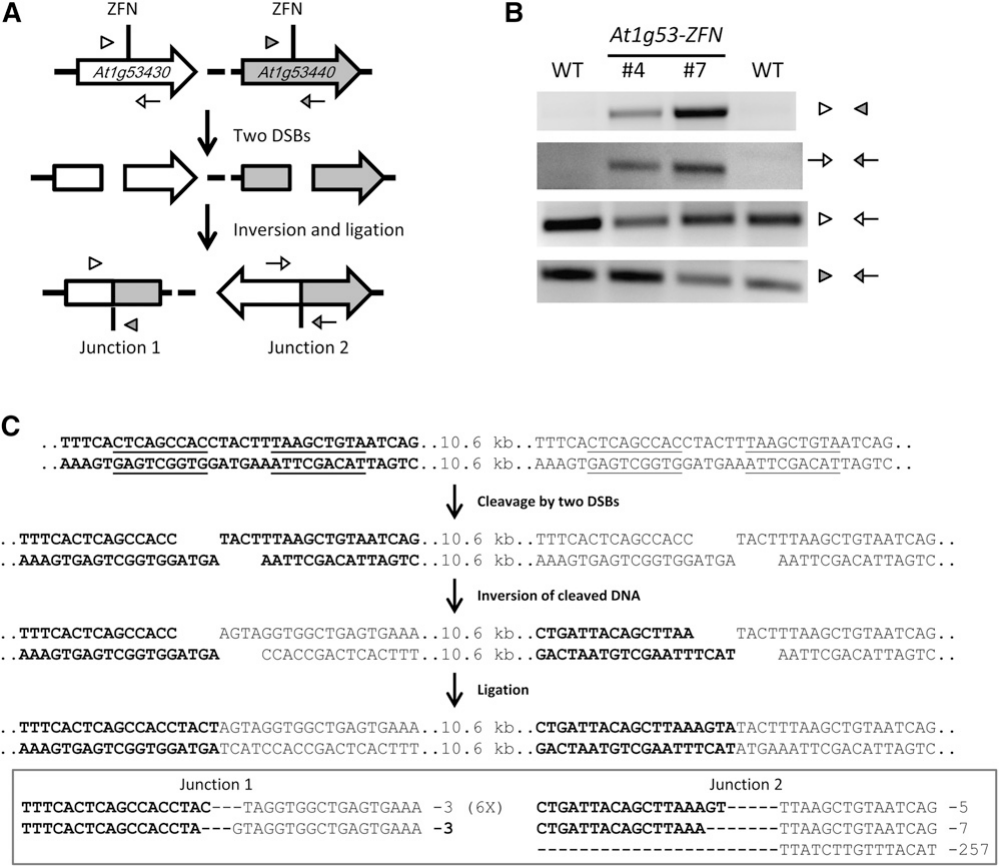
Inversion of the *At1g53430* gene cluster. (A) Schematic of the *At1g53430-At1g53440* gene cluster inversion. Positions of PCR primers for confirmation of inversions are indicated by empty or filled triangles and arrows. (B) PCR confirmation of gene cluster inversions. Two independent T2 lines were used to detect inversions; wild-type (WT) plants were used as the negative control. (C) Detailed depiction of the inversion event and DNA sequence confirmation of the inversions.

In addition to deletions, chromosomal DNA released by two DSBs could create inversions (illustrated in Figure 5A). Such inversions will have two novel junction sites, which can be detected with PCR using specific primer sets (Figure 5A). The predicted novel junctions were, in fact, detected at the At1g53430 gene cluster by PCR; DNA sequencing confirmed that the inversions occurred (Figure 5B). As anticipated, many inversion junctions had small deletions indicative of imprecise NHEJ (Figure 5C). We could also detect and confirm inversions at the *At1g70450* gene cluster (Figure S8), and these occurred at a frequency of ~0.05% as measured by digital PCR. Gene cluster inversions, therefore, appear to occur at a lower frequency than deletions.

### Possible targeted duplication of gene clusters

When two DSBs are induced in different TAGs on different chromosomes (either homologous chromosomes or sister chromatids), interchromosomal ligations of the broken DNA ends can result in deletions as well as gene cluster duplications (Figure S4A). For TAGs that only contain two genes, such as the *At1g70450* gene cluster, the duplication will create a hybrid gene (Figure S9A). We indeed detected specific products indicative of ZFN-induced duplications of the *At1g70450* gene cluster (Figure S9C), and these were also confirmed by DNA sequencing of the cloned PCR products (Figure S9D). However, if deleted DNA was circularized and retained in cells, it would give the same PCR products as depicted in Figure S9B. Because we cannot distinguish between these two outcomes, and because we do not know how long DNA circles persist in cells, it remains unclear how many of the events detected (Figure S9, C and D) truly reflect gene cluster duplications.

## Discussion

We previously reported engineering active ZFNs for six endogenous loci in Arabidopsis using the CoDA method (Sander *et al.* 2011). In this study, we successfully targeted seven additional loci with three ZFNs, each of which showed NHEJ mutagenesis activity. Two other ZFNs targeting different endogenous loci failed to show detectable activity in Arabidopsis. For these ZFNs, it remains possible that chromatin structure or epigenetic modifications impeded ZFN access to endogenous DNA targets. Our overall 60% success rate is in line with the success rate previously reported for CoDA, and our work further demonstrates the usefulness of the CoDA method for engineering active ZFNs.

We aimed to create deletions of TAGs in the Arabidopsis genome by using a single ZFN pair to target multiple genes within the cluster. However, not every gene cluster can be deleted with such a strategy. In cases in which the TAGs differ significantly in DNA sequence similarity, two different pairs of ZFNs might be required to create the desired deletion (Sollu *et al.* 2010). In addition, TALENs are good alternatives to ZFNs, because there seems to be less restriction in designing TALENs to target diverse DNA sequences (Bogdanove and Voytas 2011; Doyle *et al.* 2012; Reyon *et al.* 2012). The scale of deletions that we obtained (from a few kilobases to 9 Mb) suggests ZFNs are capable of creating very large deletions in plants. We recognize, however, that Arabidopsis plants are not likely to survive the loss of megabase pairs of chromosomal DNA. Targeted insertions created by HR were previously demonstrated by the use of ZFNs in plants (Cai *et al.* 2009). Generating deletions (such as in this study) and concomitantly creating insertions by HR may be another genome engineering approach of value for basic research and crop improvement.

Being highly homologous and repetitive, TAGs are particularly prone to change either through unequal crossover or gene conversion. This provides the opportunity to evolve new gene functions, some of which may be adaptive (Hanada *et al.* 2008; Lehti-Shiu *et al.* 2009). Our approach for making deletions and duplications in TAGs has demonstrated that we can now create novel chimeric genes which may otherwise not occur naturally. For example, we frequently detected hybrid genes due to perfect ligation of broken chromosomes after loss of the intervening DNA (Figure 3, A−C). In addition, duplication of gene clusters increases genetic redundancy and frees some gene members to evolve new functions. Tandem duplication in Arabidopsis has provided a means for adaptive evolution of *R* genes (Meyers *et al.* 2005). Our approach in creating rearrangements in the complex *RPP4* gene cluster and the two *RLK* gene clusters is thus a promising first step toward generating valuable genetic material for both molecular and evolutionary studies.

Gene cluster inversion is another consequence of our TAG-targeting approach. For TAGs encoding two genes, DNA inversion is likely to destroy the function of both gene targets. However, because inversions preserve DNA sequences (which would otherwise be lost in deletions), they may have unique applications, such as serving as templates for future genome evolution. Another application of inversions is that they may only knock out two target genes at both ends of the TAG while retaining the function of the genes in between. This is only true if there are more than two members in the TAG, such as the *RPP4* gene cluster evaluated in this study.

To recover plants with germline-transmitted deletions, we screened large T3 populations of At1g70-ZFN #2 (a total of 2539 plants) and At4g16-ZFN #12 (a total of 2322 plants) with no success (data not shown). Clearly, the frequencies of germline-transmitted deletions are much lower than the somatic frequencies. This observation is consistent with other ZFN-mediated mutagenesis studies we have conducted (unpublished data), where the frequency of somatic mutation did not directly reflect the frequency of germline mutation. Rather, we believe there is a threshold somatic mutation frequency that must be surpassed to ensure successful germline transmission. In previous work, we found that somatic mutagenesis frequencies in excess of 7% were sufficient to recover germinal mutations at high frequency (Zhang *et al.* 2010). The observed ~1% somatic deletion frequency observed here appears to be under this threshold.

It has been shown that stem cell niches in Arabidopsis are hypersensitive to DNA damage, and an even a low dose of DNA damage can trigger programmed cell death selectively in these stem cells (Fulcher and Sablowski 2009). Interestingly, programmed cell death in the shoot meristem was greatly suppressed when the DNA damage early response gene, *ATM*, was knocked out. Thus, it might be useful to use an *atm* mutant background to screen for germline deletions in plants. In addition, we recently reported that *smc6b* mutations promote NHEJ in Arabidopsis (Qi *et al.* 2013). As chromosomal breaks that lead to deletions are joined through NHEJ, it is likely that *smc6b* mutations will enhance deletion frequencies. It is also possible some deletions are deleterious to pollen and egg cells, and this impedes their transmission. Because whole plant regeneration routinely is performed from somatic cells for many species (*e.g.*, rice, maize, and tobacco), plants with deletions may be easier to achieve by regenerating somatic tissues that have been treated with sequence specific nucleases.

Note Added in Proof: See also Michelle Christian, Yiping Qi, Yong Zhang, and Daniel F. Voytas, 2013 Targeted Mutagenesis of *Arabidopsis thaliana* using Engineered TAL Effector Nucleases (TALENs) G3: Genes, Genomes, Genetics 3: 1697–1705.

## Supplementary Material

Supporting Information
