# Supplementary material for: Targeted Deletion and Inversion of Tandemly Arrayed Genes in Arabidopsis thaliana Using Zinc Finger Nucleases
Source: G3 (Bethesda). 2013 Oct 1;3(10):1707–15. doi: 10.1534/g3.113.006270 (PMC3789795; doi:10.1534/g3.113.006270)
Supplement: Supporting Information [file supp_3_10_1707__index.html]

Targeted Deletion and Inversion of Tandemly Arrayed Genes in Arabidopsis thaliana Using Zinc Finger Nucleases — Supporting Information 

# Targeted Deletion and Inversion of Tandemly Arrayed Genes in *Arabidopsis thaliana* Using Zinc Finger Nucleases

## Supporting Information for Qi *et al.*, 2013

**Files in this Data Supplement:**

- Supporting Information - Figures S1-S9 and Tables S1-S2 (PDF, 1 MB)
- Figure S1 - DNA sequences for zinc finger arrays (PDF, 297 KB)
- Figure S2 - ZFN expression entry clone-pZHY013 (PDF, 316 KB)
- Figure S3 - ZFNs that target the *ASK8* gene cluster and a lectin *RLK* gene cluster (PDF, 368 KB)
- Figure S4 - CoDA-assembled ZFNs are active in T1 plants. (PDF, 315 KB)
- Figure S5 - At1g53-ZFN activity at two targets revealed by enrichment PCR in T2 plants (PDF, 312 KB)
- Figure S6 - An active ADH1-ZFN #3 line (PDF, 411 KB)
- Figure S7 - A possible NHEJ repair mechanism using 1-bp of microhomology (PDF, 365 KB)
- Figure S8 - Inversion of the At1g70450 gene cluster (PDF, 347 KB)
- Figure S9 - Duplication of a gene cluster or circularization of deleted DNA at the At1g70450-At1g70460 locus (PDF, 377 KB)
- Table S1 - Zinc finger arrays, recognition sites and recognition helices (PDF, 299 KB)
- Table S2 - Oligos for amplifying Arabidopsis DNA (PDF, 299 KB)
