# Supplementary material for: Targeted Deletion and Inversion of Tandemly Arrayed Genes in Arabidopsis thaliana Using Zinc Finger Nucleases
Source: G3 (Bethesda). 2013 Oct 1;3(10):1707–15. doi: 10.1534/g3.113.006270 (PMC3789795; doi:10.1534/g3.113.006270)
Supplement: Supporting Information [file supp_g3.113.006270_006270SI.pdf]

G3: Genes | Genomes | Genetics

Targeted deletion and inversion of tandemly arrayed genes in *Arabidopsis thaliana* using zinc finger nucleases

Yiping Qi,[*] Xiaohong Li,[*] Yong Zhang,[*,§] Colby G Starker,[*] Nicholas J Baltes,[*] Feng Zhang,[*,1] Jeffry D Sander,[**,§§] Deepak Reyon,[**,§§,***] J Keith Joung,[**,§§] and Daniel F Voytas[*,2]

[*]Department of Genetics, Cell Biology & Development and Center for Genome Engineering, University of Minnesota, Minneapolis, Minnesota 55455, USA; [§]Department of Biotechnology, School of Life Sciences and Technology, University of Electronic Science and Technology of China, Chendu 610054, China; [**]Molecular Pathology Unit, Center for Computational and Integrative Biology, and Center for Cancer Research, Massachusetts General Hospital, Charlestown, Massachusetts 02129, USA; [§§]Department of Pathology, Harvard Medical School, Boston, Massachusetts 02115, USA; [***]Department of Genetics, Development and Cell Biology, Interdepartmental Graduate Program in Bioinformatics & Computational Biology, Iowa State University, Ames, Iowa 50011, USA.

[1]Current address: Cellectis Plant Sciences, 1000 Westgate Drive, Suite 136, St. Paul, MN 55114, USA.
[2]Corresponding author.

DOI: 10.1534/g3.113.006270

> At1g53-ZF_Left
GAAAAAAATCTAGACCCGGGGAGCGCCCCTTCCAGTGTCGCATTTGCATGCGGAACTTTTCGAAACATTCTAACTTGACCCGTCATACCC
GTACTCATACCGGTGAAAAACCGTTTCAGTGTCGGATCTGTATGCGAAATTTCTCCCAGCGTTCTGACTTGACCCGTCATCTACGTACGC
ACACCGGCGAGAAGCCATTCCAATGCCGAATATGCATGCGCAACTTCAGTCGTCCAGACGCATTGCCACGTCACCTAAAAACCCACCTG
AGGGGATCCAAGAAGGA

> At1g53-ZF_right
GAAAAAAATCTAGACCCGGGGAGCGCCCCTTCCAGTGTCGCATTTGCATGCGGAACTTTTCGCAGCAGTCTTCTTTGTTGCGTCATACCC
GTACTCATACCGGTGAAAAACCGTTTCAGTGTCGGATCTGTATGCGAAATTTCTCCCAGCGTTCTGACTTGACCCGTCATCTACGTACGC
ACACCGGCGAGAAGCCATTCCAATGCCGAATATGCATGCGCAACTTCAGTCAGCGTGGTAACTTGAACATGCACCTAAAAACCCACCTG
AGGGGATCCAAGAAGGA

> At1g70-ZF_left
GAAAAAAATCTAGACCCGGGGAGCGCCCCTTCCAGTGTCGCATTTGCATGCGGAACTTTTCGATGAAAAACACCTTGACCCGTCATACC
CGTACTCATACCGGTGAAAAACCGTTTCAGTGTCGGATCTGTATGCGAAATTTCTCCCAGCGTTCTGACTTGACCCGTCATCTACGTACG
CACACCGGCGAGAAGCCATTCCAATGCCGAATATGCATGCGCAACTTCAGTCAGCGTGGTAACTTGAACATGCACCTAAAAACCCACCT
GAGGGGATCCAAGAAGGA

> At1g70-ZF_right
GAAAAAAATCTAGACCCGGGGAGCGCCCCTTCCAGTGTCGCATTTGCATGCGGAACTTTTCGGACCCATCTAACTTGATTCGTCATACCC
GTACTCATACCGGTGAAAAACCGTTTCAGTGTCGGATCTGTATGCGAAATTTCTCCCGTACCGACACCTTGGCACGTCATCTACGTACGC
ACACCGGCGAGAAGCCATTCCAATGCCGAATATGCATGCGCAACTTCAGTCAGGGTGGTGCATTGCAGCGTCACCTAAAAACCCACCTG
AGGGGATCCAAGAAGGA

> At4g16-ZF_left
GAAAAAAATCTAGACCCGGGGAGCGCCCCTTCCAGTGTCGCATTTGCATGCGGAACTTTTCGCAGGCATCTAACTTGACCCGTCATACC
CGTACTCATACCGGTGAAAAACCGTTTCAGTGTCGGATCTGTATGCGAAATTTCTCCCAGCAGACCAACTTGACCCGTCATCTACGTACG
CACACCGGCGAGAAGCCATTCCAATGCCGAATATGCATGCGCAACTTCAGTCAGACCAACAACTTGAACCGTCACCTAAAAACCCACCT
GAGGGGATCCAAGAAGGA

> At4g16-ZF_right
GAAAAAAATCTAGACCCGGGGAGCGCCCCTTCCAGTGTCGCATTTGCATGCGGAACTTTTCGGACAACGCACATTTGGCACGTCATACC
CGTACTCATACCGGTGAAAAACCGTTTCAGTGTCGGATCTGTATGCGAAATTTCTCCGACTCTTCTGTTTTGCGTCGTCATCTACGTACGC
ACACCGGCGAGAAGCCATTCCAATGCCGAATATGCATGCGCAACTTCAGTCAGTCTACCTCTTTGCAGCGTCACCTAAAAACCCACCTGA
GGGGATCCAAGAAGGA

> At3g21-ZF_left
GAAAAAAATCTAGACCCGGGGAGCGCCCCTTCCAGTGTCGCATTTGCATGCGGAACTTTTCGAAACGTCAGCATTTGGAATATCATACCC
GTACTCATACCGGTGAAAAACCGTTTCAGTGTCGGATCTGTATGCGAAATTTCTCCCAGCGTTCTGACTTGACCCGTCATCTACGTACGC
ACACCGGCGAGAAGCCATTCCAATGCCGAATATGCATGCGCAACTTCAGTCATGGTCATCGTTTGAAAACCCACCTAAAAACCCACCTGA
GGGGATCCAAGAAGGA

> At3g21-ZF_right
GAAAAAAATCTAGACCCGGGGAGCGCCCCTTCCAGTGTCGCATTTGCATGCGGAACTTTTCGTTGCGTACCTCTTTGGTTCGTCATACCC
GTACTCATACCGGTGAAAAACCGTTTCAGTGTCGGATCTGTATGCGAAATTTCTCCGACTCTTCTGTTTTGCGTCGTCATCTACGTACGCA
CACCGGCGAGAAGCCATTCCAATGCCGAATATGCATGCGCAACTTCAGTTTGTCTACCAACTTGACCCGTCACCTAAAAACCCACCTGAG
GGGATCCAAGAAGGA

> At5g01-ZF_left
GAAAAAAATCTAGACCCGGGGAGCGCCCCTTCCAGTGTCGCATTTGCATGCGGAACTTTTCGCGTCCATCTAAATTGGTTTTGCATACCC
GTACTCATACCGGTGAAAAACCGTTTCAGTGTCGGATCTGTATGCGAAATTTCTCCTTGAAAGAACATTTGACCCGTCATCTACGTACGCA
CACCGGCGAGAAGCCATTCCAATGCCGAATATGCATGCGCAACTTCAGTCAGTCTCAGCATTTGGTTCGTCACCTAAAAACCCACCTGAG
GGGATCCAAGAAGGA

> At5g01-ZF_right
GAAAAAAATCTAGACCCGGGGAGCGCCCCTTCCAGTGTCGCATTTGCATGCGGAACTTTTCGCAGGCATCTAACTTGACCCGTCATACC
CGTACTCATACCGGTGAAAAACCGTTTCAGTGTCGGATCTGTATGCGAAATTTCTCCCAGCAGACCAACTTGACCCGTCATCTACGTACG
CACACCGGCGAGAAGCCATTCCAATGCCGAATATGCATGCGCAACTTCAGTAAAAACGTTTCTTTGACCCATCACCTAAAAACCCACCTG
AGGGGATCCAAGAAGGA

Figure S1 DNA sequences for zinc finger arrays. DNA sequences for ten zinc finger arrays of five pairs of ZFNs are shown. The restriction enzyme sites (XbaI and BamHI) for subcloning of the zinc finger arrays into expression vectors are marked in red and blue, respectively.

A

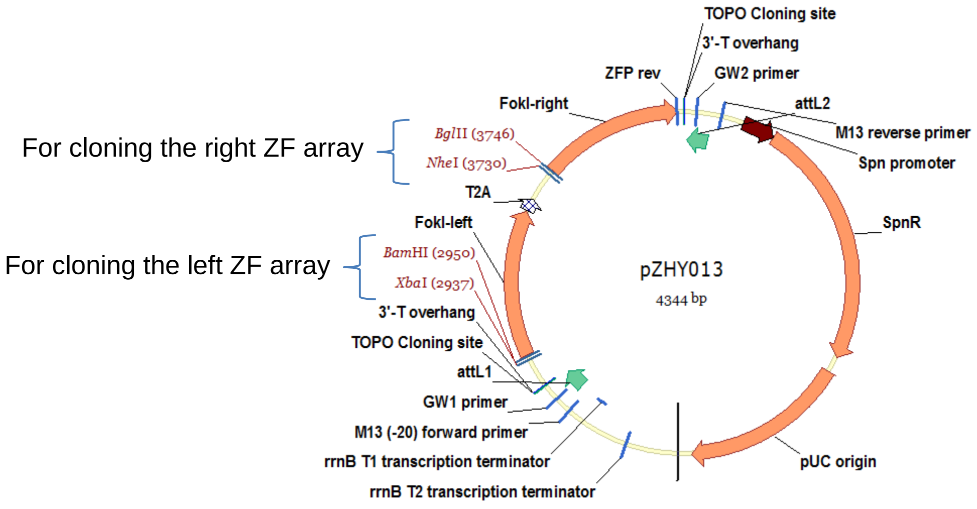

B

>pZHY013
aagggcgaattcgacccagctttcttgtacaaagttggcattataaaaaataattgctcatcaatttgttgcaacgaacaggtcactatcagtcaaaataaaatcattatttgccatccagctga tatcccctatagtgagtcgtattacatggtc
atagctgtttcctggcagctctggcccgtgtctcaaaatctctgatgttacattgcacaagataaaaatatatcatcatgcctcctctggaccagccaggacagaaatgcctcgacttcgctgctgcccaaggttgccgggtgacgcacaccg
tggaaacggatgaaggcacgaacccagtggacataagcctgttcggttcgtaagctgtaatgcaagtagcgtatgcgctcacgcaactggtccagaaccttgaccgaacgcagcggtggtaacgg cgcagtggcggttttcatggcttg
ttatgactgtttttttggggtacagtctatgcctcgggcatccaagcagcaagcgcgttacgccgtgggtcgatgtttgatgttatggagcagcaacgatgttacgcagcagggcagtcgccta aacaaagttaaacatcatgagggaag
cggtgatcgccgaagtatcgactcaactatcagaggtagttggcgtcatcgagcgccatctcgaaccgacgttgctggccgtacatttgtacggctccgcagtggatggcggcctgaagccacac agtgatattgatttgctggttacggtg
accgtaaggcttgatgaaacaacgcggcgagctttgatcaacgaccttttggaaacttcggcttcccctggagagagcgagattctccgcgctgtagaagtcaccattgttgtgcacgacgacat cattccgtggcgttatccagctaagcg
cgaactgcaatttggagaatggcagcgcaatgacattcttgcaggtatcttcgagccagccacgatcgacattgatctggctatcttgctgacaaaagcaagagaacatagcgttgccttggtag gtccagcggcggaggaactctttgat
ccggttcctgaacaggatctatttgaggcgctaaatgaaaccttaacgctatggaactcgccgcccgactgggctggcgatgagcgaaatgtagtgcttacgttgtcccgcatttggtacagcgc agtaaccggcaaaatcgcgccgaa
ggatgtcgctgccgactgggcaatggagcgcctgccggcccagtatcagcccgtcatacttgaagctagacaggcttatcttggacaagaagaagatcgcttggcctcgcgcgcagatcagttgg aagaatttgtccactacgtgaaag
gcgagatcaccaaggtagtcggcaaataaccctcgagccacccatgaccaaaatcccttaacgtgagttacgcgtcgttccactgagcgtcagaccccgtagaaaagatcaaaggatcttcttga gatcctttttttctgcgcgtaatctgct
gcttgcaaacaaaaaaaccaccgctaccagcggtggtttgtttgccggatcaagagctaccaactctttttccgaaggtaactggcttcagcagagcgcagataccaaatactgtccttctagtg tagccgtagttaggccaccacttcaag
aactctgtagcaccgcctacatacctcgctctgctaatcctgttaccagtggctgctgccagtggcgataagtcgtgtcttaccgggttggactcaagacgatagttaccggataaggcgcagcg gtcgggctgaacggggggttcgtgca
cacagcccagcttggagcgaacgacctacaccgaactgagatacctacagcgtgagcattgagaaagcgccacgcttcccgaagggagaaaggcggacaggtatccggtaagcggcagggtcgga acaggagagcgcacga
gggagcttccagggggaaacgcctggtatctttatagtcctgtcgggtttcgccacctctgacttgagcgtcgatttttgtgatgctcgtcaggggggcggagcctatggaaaaacgccagcaac gcggcctttttacggttcctggccttttgct
ggccttttgctcacatgttctttcctgcgttatcccctgattctgtggataaccgtattaccgcctttgagtgagctgataccgctcgccgcagccgaacgaccgagcgcagcgagtcagtgagc gaggaagcggaagagcgcccaatacg
caaaccgcctctccccgcgcgttggccgattcattaatgcagctggcacgacaggtttcccgactggaaagcgggcagtgagcgcaacgcaattaatacgcgtaccgctcgccaggaagagtttg tagaaacgcaaaaaggccatc
cgtcaggatggccttctgcttagtttgatgcctggcagtttatggcgggcgtcctgcccgccaccctccgggccgttgcttcacaacgttcaaatccgctcccggcggatttgtcctactcagga gagcgttcaccgacaaacaacagataaa
acgaaaggcccagtcttccgactgagcctttcgttttatttgatgcctggcagttccctactctcgcgttaacgcttgcatggatgttttcccagtcacgacgttgtaaaacgacggccagtctt aagctcgggccccaaataatgattttattttgac
tgatagtgacctgttcgttgcaacaaattgatgagcaatgcttttttataatgccaactttgtacaaaaaagcaggctccgaattcgcccttcaccatggattataaggatcacgatggcgacta caaggaccacgatattgactacaaagac
gatgacgataaaatggctcctaagaaaaagcgcaaagtcggtatccatggcgttccctctagataacgcaggatcccagctggtgaagtccgagctggaagaaaaaaagagcgagctgcgccaca agctcaagtacgtgccccac
gagtacatcgagctgatcgagatcgcccgcaacagcacccaagaccgcatcctggagatgaaagtgatggagttcttcatgaaggtgtacggctaccgcggcaagcacctgggcggctcccgcaa gcccgatggcgccatctaca
ccgtgggctcccccatcgactatggcgtcattgtcgacaccaaggcctactccggcggctacaacttacccatcggtcaggccgacgagatgcaacgctacgtgaaggagaaccagacccgcaat aagcacattaatcccaacgag
tggtggaaggtgtacccctcctccgtgaccgagttcaaattcctgttcgtgtccggccacttcaagggcaattataaggcccaactgacccgcctgaaccacaagaccaactgcaacggcgccgt gctgtccgtggaggaactgctgatc
ggcggcgagatgatcaaggctggtaccctgaccctggaagaggtgcgccgcaagttcaacaatggtgaaatcaatttcaggtccggcggcggagagggcagaggaagtcttctaacatgcggtga cgtggaggagaatcccggcc
ctaggatggactacaaagaccatgacggtgattataaagatcatgacatcgattacaaggatgacgatgacaagatggcccccaagaagaagaggaaggtgggcattcacggggtgccggctagc tagtaagcttagatctcaact
agtcaaaagtgaactggaggagaagaaatctgaacttcgtcataaattgaaatatgtgcctcatgaatatattgaattaattgaaattgccagaaattccactcaggatagaattcttgaaatga aggtaatggaatttttatgaaagtttatg
gatatagaggtaaacatttgggtggatcaaggaaaccggacggagcaatttatactgtcggatctcctattgattacggtgtgatcgtggatactaaagcttatagcggaggttataatctgcca attggccaagcagatgaaatggagcg
atatgtcgaagaaaatcaaacacgaaacaaacatctcaaccctaatgaatggtggaaagtctatccatcttctgtaacggaatttaagtttttatttgtgagtggtcactttaaaggaaactaca aagctcagcttacacgattaaatcatatc
actaattgtaatggagctgttcttagtgtagaagagcttttaattggtggagaaatgattaaagccggcacattaaccttagaggaagtgagacggaaatttaataacggcgagataaactttta atag

Figure S2 ZFN expression entry clone-pZHY013. (A) Map of the pZHY013 entry clone. The restriction enzyme sites for cloning zinc finger arrays are marked. (B) Full sequence of the pZHY013 entry clone.

A

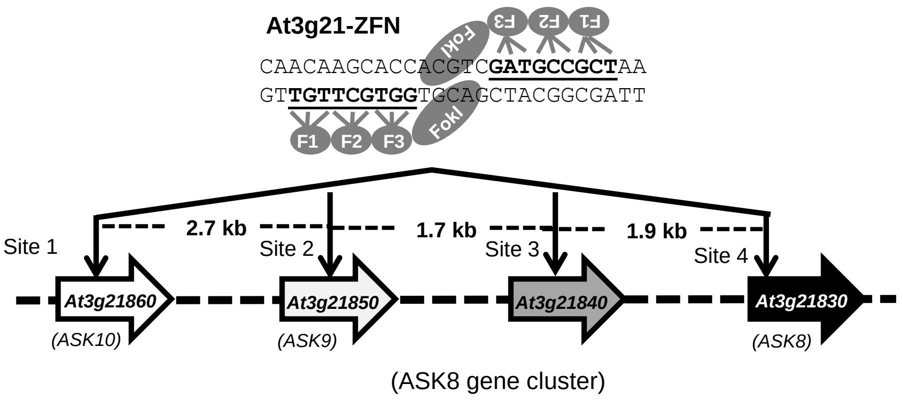

B

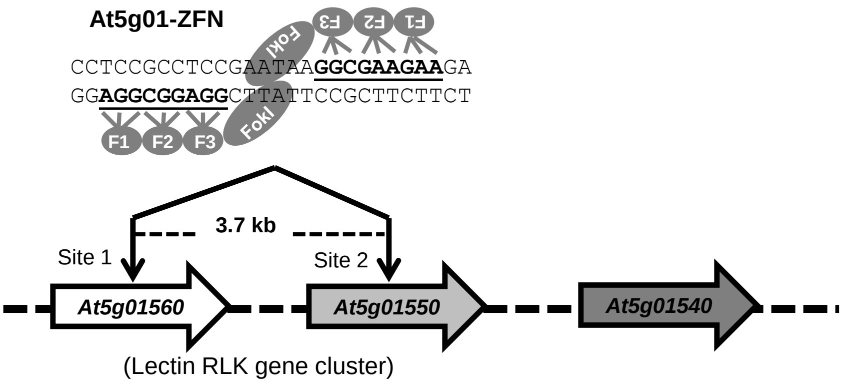

Figure S3 ZFNs that target the *ASK8* gene cluster and a lectin *RLK* gene cluster. (A) The At3g21-ZFN targets all four members of the *ASK8* gene cluster. (B) The At5g01-ZFN targets two genes in a lectin *RLK* gene cluster. Cartoons illustrate the ZFN pairs, and the DNA recognition triplets are indicated. The zinc finger binding sequences are underlined and the distance between cleavage sites is shown.

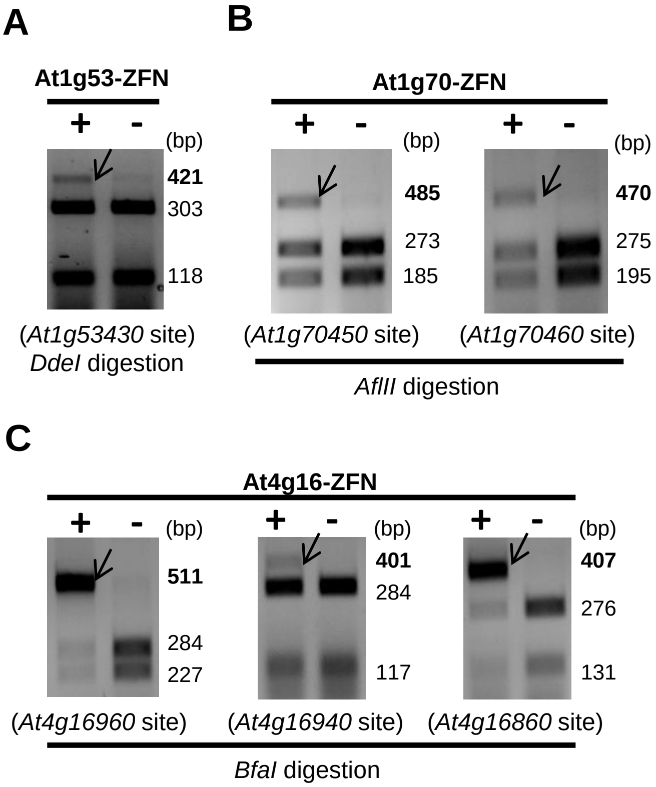

Figure S4 CoDA-assembled ZFNs are active in T1 plants. (A) At1g53-ZFN's activity is detected at *At1g53430*. (B) At1g70-ZFN's activity is detected at both *At1g70450* and *At1g70460*. (C) At4g16-ZFN's activity is detected at *At4g16960, At4g16940* and *At4g16860.* Activity of ZFNs was measured by enrichment PCR using the restriction enzymes shown in each panel. The uncut bands represent ZFN-induced mutations and are indicated by arrows. Bulked estradiol-treated T1 transgenic plants or wild type plants were compared.

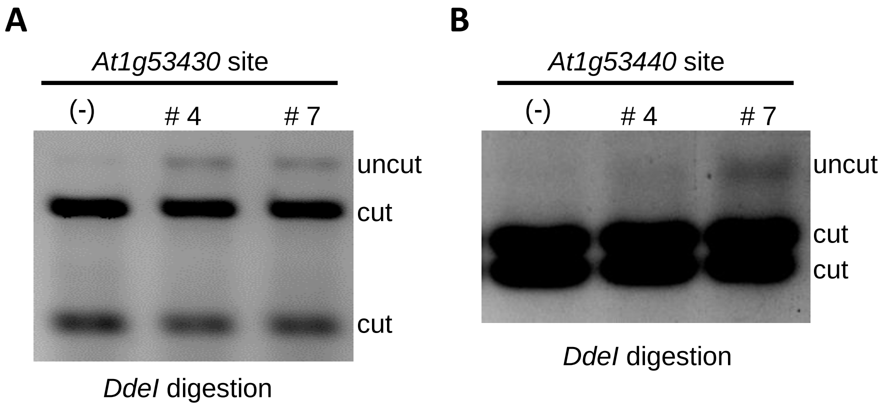

Figure S5 At1g53-ZFN activity at two targets revealed by enrichment PCR in T2 plants. (A) Enrichment PCR detection of ZFN activity at the *At1g53430* locus in two T2 transgenic populations. (B) Enrichment PCR detection of ZFN activity at the *At1g53440* locus in two T2 transgenic plant populations.

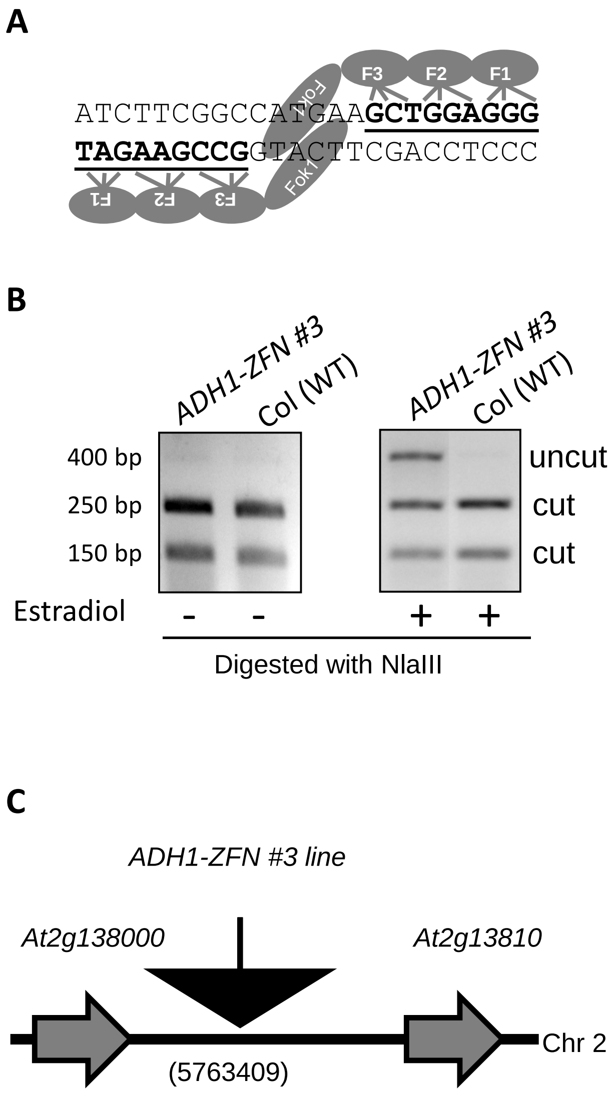

Figure S6 An active ADH1-ZFN #3 line. (A) Schematic of the ADH1-ZFN and its target site. (B) ADH1-ZFN activity is highly estradiol-inducible. Mutagenesis activity, as reflected by the uncut band, was detected by PCR and digestion (C) Precise location of the transgene in *ADH1-ZFN* #3 line as mapped by TAIL-PCR.

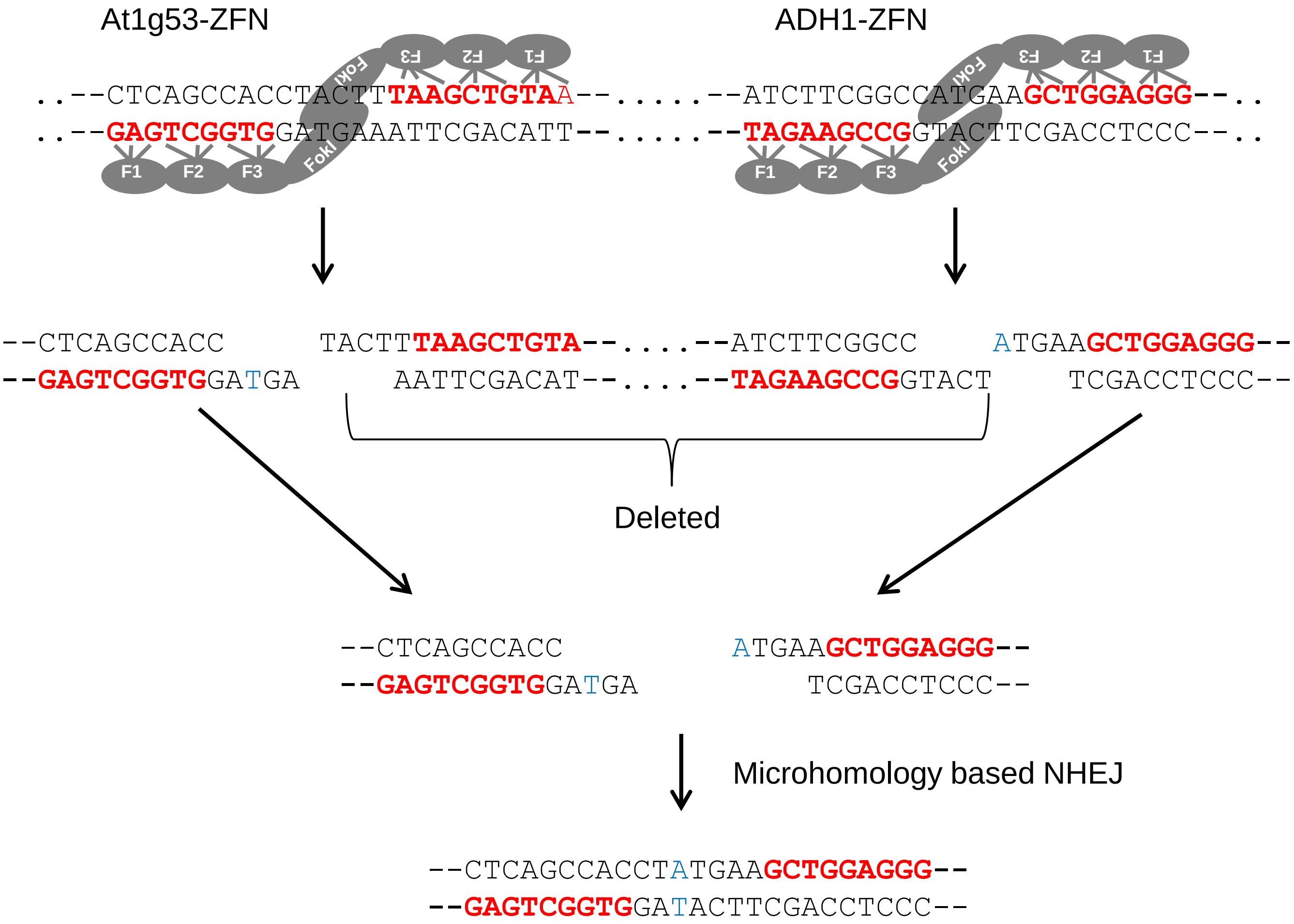

Figure S7 A possible NHEJ repair mechanism using 1-bp of microhomology. The process that leads to a common ligation product is depicted. The ZFN binding sites are shown in red and the 1 nt of likely microhomology is marked in blue.

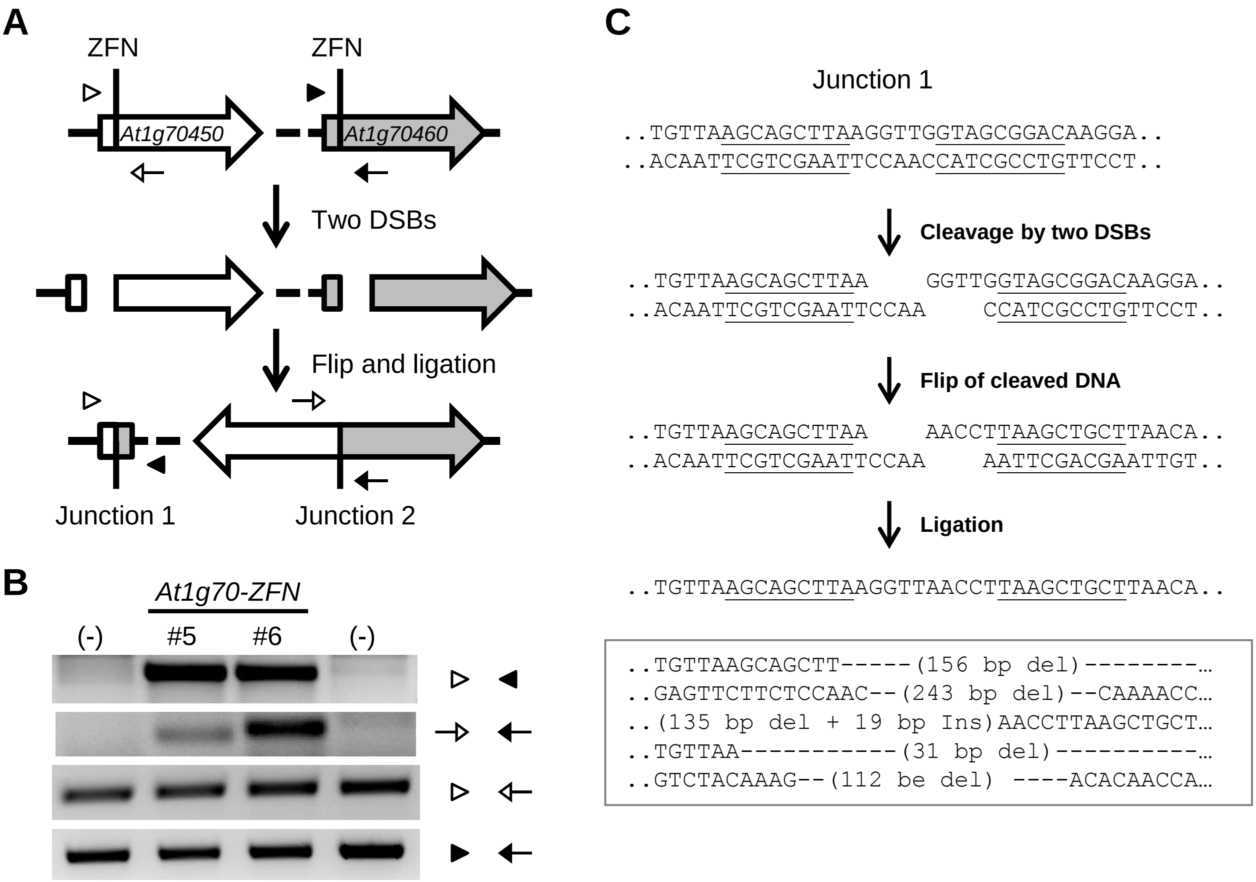

Figure S8 Inversion of the At1g70450 gene cluster. (A) Schematic of the *At1g70450* gene cluster inversion. Positions of PCR primers for confirming inversions are indicated by empty or filled triangles and arrows. (B) PCR confirmation of gene cluster inversions. (C) DNA sequence confirmation of inversions.

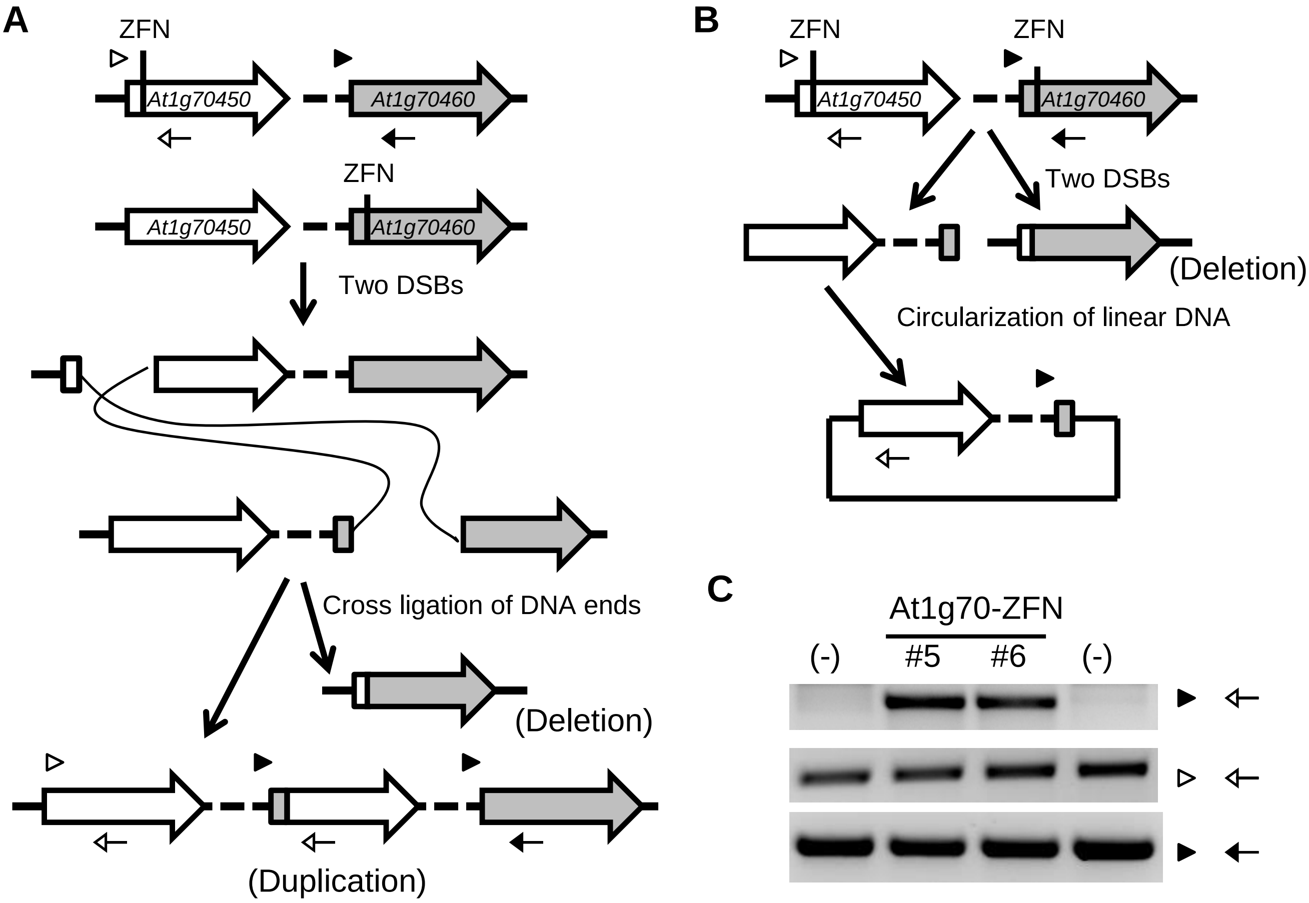

D

```
TGTTAAGCAGCTTAAGGTTGGTAGCGGACAAGGT(PL)
TGTTAAGCAGCTTAAGGTTGGTAGCGGACAAGGT(PL,20X)
TGTTAAGCAGCTTAAGGgTTGGTAGCGGACAAGG +1
TGTTAAGCAGCTTAAGGTTtGGTAGCGGACAAGG +1 (6X)
TGTTAAG-AGCTTAAGGTTGGTAGCGGACAAGGT -1
TGTTAAGCAGCTTAAGG---GTAGCGGACAAGGT -3
TGTTAAGCAGCTTAAGGTT---AGCGGACAAGGT -3
TGTTAAGCAGCTTAA---TGGTAGCGGACAAGGT -3
TGTTAAG--------------------------- -42
-----------------------GCGGACAAGGT -48
```

Figure S9 Duplication of a gene cluster or circularization of deleted DNA at the At1g70450-At1g70460 locus. (A) Schematic of the *At1g70450* gene cluster duplication. (B) Schematic of circularization of deleted DNA. (C) PCR confirmation of possible gene cluster duplications. (D) DNA sequence data from clones indicative of possible gene cluster duplications.

Table S1 Zinc finger arrays, recognition sites and recognition helices.

Zinc finger arrays	Recognition sites and recognition helix amino acid sequences		
	F1	F2	F3
At1g53-ZF_left	GAG	GCT	GTG
	KHSNLTR	QRSDLTR	RPDALPR
At1g53-ZF_right	GTA	GCT	TAA
	QQSSLLR	QRSDLTR	QRGNLNM
At1g70-ZF_left	GCT	GCT	TAA
	MKNTLTR	QRSDLTR	QRGNLNM
At1g70-ZF_right	GAC	GCG	GTA
	DPSNLIR	RTDTLAR	QGGALQR
At4g16-ZF_left	GAA	GAA	GAA
	QASNLTR	QQTNLTR	QTNNLNR
At4g16-ZF_right	GGA	GCC	GTA
	DNAHLAR	DSSVLRR	QSTSLQR
At3g21-ZF_left	TGT	GCT	GGT
	KRQHLEY	QRSDLTR	HGHRLKT
At3g21-ZF_right	GCT	GCC	GAT
	LRTSLVR	DSSVLRR	LSTNLTR
At5g01-ZF_left	GGA	GGC	GGA
	RPSKLVL	LKEHLTR	QSQHLVR
At5g01-ZF_right	GAA	GAA	GGC
	QASNLTR	QQTNLTR	KNVSLTH

Table S2   Oligos for amplifying Arabidopsis DNA

Oligo name	Oligo sequence	Purpose	Note
At1g53430-F2	CTgtaagcaaaactaactaaccac	For detection of mutations at At1g53430 site	
At1g53430-R2	ctcacGTTTAGCATCTTCTGGACA	For detection of mutations at At1g53430 site and gene cluster inversions	Designated as an open arrow in Fig. S5A and B
At1g53440-F1	tagatgatatttttaaccgtgac	For detection of gene cluster inversions and large chromosomal deletions	Designated as F2 in Fig. 4A and as a filled, tailess arrow in Fig. 5A and B
At1g53440-F2	tattcggtatcatcaaggtca	For detection of mutations at At1g53440 site	
At1g53440-R2	TTCTTAAGCACCATTGGACActac	For detection of mutations at At1g53440 site	
At1g53430-F1	ATTGGTTCCATGAGTGAGC	For detection of gene cluster deletions, inversions and large chromosomal deletions	Designated as F1 in Fig. 3A and Fig. 4A, and as an open tailess arrow in Fig. 5A and B
At1g53440-R3	aagggcttctttttttcaag	For detection of gene cluster deletions and inversions	Designated as R2 in Fig. 3A and as a filled arrow in Fig. 5A and B
At1g70450-F3	TTCTTCTCCAACAGCACCGTCAG	For detection of mutations at At1g70450 site and gene cluster deletions	Designated as F1 in Fig. 3B
At1g70450-R2	CACTGGCCTACCCTTCCctgtc	For detection of mutations at At1g70450 site and gene cluster inversions	Designated as an open arrow in Fig. S8A and B
At1g70450-R3	GGTGACctgcaaaacaagataaat	For detection of gene cluster duplications	Designated as an open arrow in Fig. S9
At1g70460-F	TACTCTGGTCCTGGTGGTTACAAT	For detection of gene cluster duplications	Designated as a tailess filled arrow in Fig. S9
At1g70460-F3	GAGGAGGAGGTTATACACGGTCAG	For detection of mutations at At1g70460 site	
At1g70460-R2	AGTACTGGCCTTCCCTTCCctatc	For detection of mutations at At1g70460 site and gene cluster inversions	Designated as an filled arrow in Fig. S8A and B
At1g70460-R3	tgcaaaacaaaacaaaaacataca	For detection of gene cluster deletions	Designated as R2 in Fig. 3B
At4g16960-F	gtcttgttaggtggtttgatgtta	For detection of NHEJ-mediated mutagenesis	
At4g16960/940-R	CCATTTGATCCAAGTCTTTG	For detection of mutations at At4g16960 and At4g16940 sites	
At4g16940-F	agcaccacctcagccccatac	For detection of mutations at At4g16960 site and gene cluster deletions	Designated as F2 in Fig. 3C
At4g16860/950-F	tggagggaaggaagacgaagtt	For detection of mutations at At4g16860 site	
At4g16860-R	ATTTGTTCCCCTTTCTTGTA	For detection of mutations at At4g16860 site and gene cluster deletions	
At4g16960-F2	tctgtatcatattagtttagttcg	For detection of gene cluster deletions	Designated as F1 in Fig. 3C
At4g16940-R2	aaagagaataacacagatttattt	For detection of gene cluster deletions	Designated as R2 in Fig. 3C
ADH1F	TCGAGGAAGTGGAGGTTGCT	For detection of mutations at the ADH1 site	
ADH1R2	TGGCTGAAGATCAGTCACTCC	For detection of mutations at the ADH1 site and large chromosomal deletions	Designated as R in Fig. 4A
