# Supplementary material for: Targeted Deletion and Inversion of Tandemly Arrayed Genes in Arabidopsis thaliana Using Zinc Finger Nucleases
Source: G3 (Bethesda). 2013 Oct 1;3(10):1707–15. doi: 10.1534/g3.113.006270 (PMC3789795; doi:10.1534/g3.113.006270)
Supplement: Supporting Information [file supp_g3.113.006270_FigureS1.pdf]

> At1g53-ZF_Left
GAAAAAAATCTAGACCCGGGGAGCGCCCCTTCCAGTGTCGCATTTGCATGCGGAACTTTTCGAAACATTCTAACTTGACCCGTCATACCC
GTACTCATACCGGTGAAAAACCGTTTCAGTGTCGGATCTGTATGCGAAATTTCTCCCAGCGTTCTGACTTGACCCGTCATCTACGTACGC
ACACCGGCGAGAAGCCATTCCAATGCCGAATATGCATGCGCAACTTCAGTCGTCCAGACGCATTGCCACGTCACCTAAAAACCCACCTG
AGGGGATCCAAGAAGGA

> At1g53-ZF_right
GAAAAAAATCTAGACCCGGGGAGCGCCCCTTCCAGTGTCGCATTTGCATGCGGAACTTTTCGCAGCAGTCTTCTTTGTTGCGTCATACCC
GTACTCATACCGGTGAAAAACCGTTTCAGTGTCGGATCTGTATGCGAAATTTCTCCCAGCGTTCTGACTTGACCCGTCATCTACGTACGC
ACACCGGCGAGAAGCCATTCCAATGCCGAATATGCATGCGCAACTTCAGTCAGCGTGGTAACTTGAACATGCACCTAAAAACCCACCTG
AGGGGATCCAAGAAGGA

> At1g70-ZF_left
GAAAAAAATCTAGACCCGGGGAGCGCCCCTTCCAGTGTCGCATTTGCATGCGGAACTTTTCGATGAAAAACACCTTGACCCGTCATACC
CGTACTCATACCGGTGAAAAACCGTTTCAGTGTCGGATCTGTATGCGAAATTTCTCCCAGCGTTCTGACTTGACCCGTCATCTACGTACG
CACACCGGCGAGAAGCCATTCCAATGCCGAATATGCATGCGCAACTTCAGTCAGCGTGGTAACTTGAACATGCACCTAAAAACCCACCT
GAGGGGATCCAAGAAGGA

> At1g70-ZF_right
GAAAAAAATCTAGACCCGGGGAGCGCCCCTTCCAGTGTCGCATTTGCATGCGGAACTTTTCGGACCCATCTAACTTGATTCGTCATACCC
GTACTCATACCGGTGAAAAACCGTTTCAGTGTCGGATCTGTATGCGAAATTTCTCCCGTACCGACACCTTGGCACGTCATCTACGTACGC
ACACCGGCGAGAAGCCATTCCAATGCCGAATATGCATGCGCAACTTCAGTCAGGGTGGTGCATTGCAGCGTCACCTAAAAACCCACCTG
AGGGGATCCAAGAAGGA

> At4g16-ZF_left
GAAAAAAATCTAGACCCGGGGAGCGCCCCTTCCAGTGTCGCATTTGCATGCGGAACTTTTCGCAGGCATCTAACTTGACCCGTCATACC
CGTACTCATACCGGTGAAAAACCGTTTCAGTGTCGGATCTGTATGCGAAATTTCTCCCAGCAGACCAACTTGACCCGTCATCTACGTACG
CACACCGGCGAGAAGCCATTCCAATGCCGAATATGCATGCGCAACTTCAGTCAGACCAACAACTTGAACCGTCACCTAAAAACCCACCT
GAGGGGATCCAAGAAGGA

> At4g16-ZF_right
GAAAAAAATCTAGACCCGGGGAGCGCCCCTTCCAGTGTCGCATTTGCATGCGGAACTTTTCGGACAACGCACATTTGGCACGTCATACC
CGTACTCATACCGGTGAAAAACCGTTTCAGTGTCGGATCTGTATGCGAAATTTCTCCGACTCTTCTGTTTTGCGTCGTCATCTACGTACGC
ACACCGGCGAGAAGCCATTCCAATGCCGAATATGCATGCGCAACTTCAGTCAGTCTACCTCTTTGCAGCGTCACCTAAAAACCCACCTGA
GGGGATCCAAGAAGGA

> At3g21-ZF_left
GAAAAAAATCTAGACCCGGGGAGCGCCCCTTCCAGTGTCGCATTTGCATGCGGAACTTTTCGAAACGTCAGCATTTGGAATATCATACCC
GTACTCATACCGGTGAAAAACCGTTTCAGTGTCGGATCTGTATGCGAAATTTCTCCCAGCGTTCTGACTTGACCCGTCATCTACGTACGC
ACACCGGCGAGAAGCCATTCCAATGCCGAATATGCATGCGCAACTTCAGTCATGGTCATCGTTTGAAAACCCACCTAAAAACCCACCTGA
GGGGATCCAAGAAGGA

> At3g21-ZF_right
GAAAAAAATCTAGACCCGGGGAGCGCCCCTTCCAGTGTCGCATTTGCATGCGGAACTTTTCGTTGCGTACCTCTTTGGTTCGTCATACCC
GTACTCATACCGGTGAAAAACCGTTTCAGTGTCGGATCTGTATGCGAAATTTCTCCGACTCTTCTGTTTTGCGTCGTCATCTACGTACGCA
CACCGGCGAGAAGCCATTCCAATGCCGAATATGCATGCGCAACTTCAGTTTGTCTACCAACTTGACCCGTCACCTAAAAACCCACCTGAG
GGGATCCAAGAAGGA

> At5g01-ZF_left
GAAAAAAATCTAGACCCGGGGAGCGCCCCTTCCAGTGTCGCATTTGCATGCGGAACTTTTCGCGTCCATCTAAATTGGTTTTGCATACCC
GTACTCATACCGGTGAAAAACCGTTTCAGTGTCGGATCTGTATGCGAAATTTCTCCTTGAAAGAACATTTGACCCGTCATCTACGTACGCA
CACCGGCGAGAAGCCATTCCAATGCCGAATATGCATGCGCAACTTCAGTCAGTCTCAGCATTTGGTTCGTCACCTAAAAACCCACCTGAG
GGGATCCAAGAAGGA

> At5g01-ZF_right
GAAAAAAATCTAGACCCGGGGAGCGCCCCTTCCAGTGTCGCATTTGCATGCGGAACTTTTCGCAGGCATCTAACTTGACCCGTCATACC
CGTACTCATACCGGTGAAAAACCGTTTCAGTGTCGGATCTGTATGCGAAATTTCTCCCAGCAGACCAACTTGACCCGTCATCTACGTACG
CACACCGGCGAGAAGCCATTCCAATGCCGAATATGCATGCGCAACTTCAGTAAAAACGTTTCTTTGACCCATCACCTAAAAACCCACCTG
AGGGGATCCAAGAAGGA

Figure S1 DNA sequences for zinc finger arrays. DNA sequences for ten zinc finger arrays of five pairs of ZFNs are shown. The restriction enzyme sites (XbaI and BamHI) for subcloning of the zinc finger arrays into expression vectors are marked in red and blue, respectively.
