# Supplementary material for: Targeted Deletion and Inversion of Tandemly Arrayed Genes in Arabidopsis thaliana Using Zinc Finger Nucleases
Source: G3 (Bethesda). 2013 Oct 1;3(10):1707–15. doi: 10.1534/g3.113.006270 (PMC3789795; doi:10.1534/g3.113.006270)
Supplement: Supporting Information [file supp_g3.113.006270_FigureS3.pdf]

A

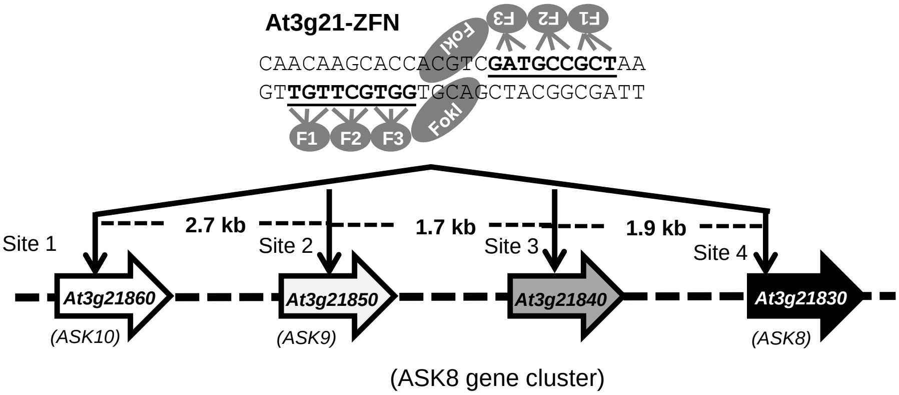

B

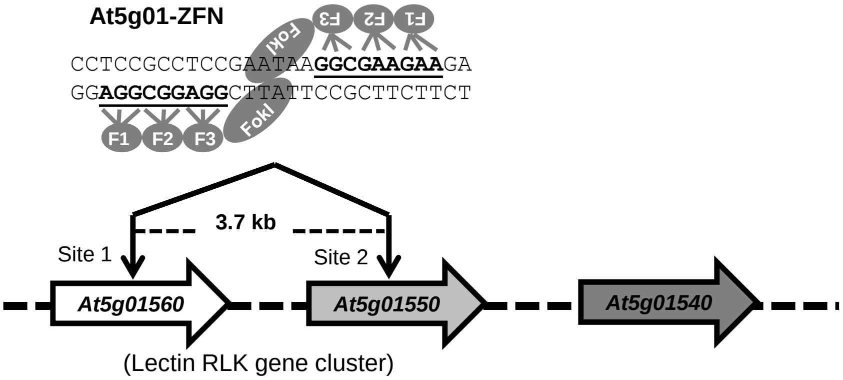

Figure S3 ZFNs that target the *ASK8* gene cluster and a lectin *RLK* gene cluster. (A) The At3g21-ZFN targets all four members of the *ASK8* gene cluster. (B) The At5g01-ZFN targets two genes in a lectin *RLK* gene cluster. Cartoons illustrate the ZFN pairs, and the DNA recognition triplets are indicated. The zinc finger binding sequences are underlined and the distance between cleavage sites is shown.
