# Supplementary material for: Targeted Deletion and Inversion of Tandemly Arrayed Genes in Arabidopsis thaliana Using Zinc Finger Nucleases
Source: G3 (Bethesda). 2013 Oct 1;3(10):1707–15. doi: 10.1534/g3.113.006270 (PMC3789795; doi:10.1534/g3.113.006270)
Supplement: Supporting Information [file supp_g3.113.006270_FigureS4.pdf]

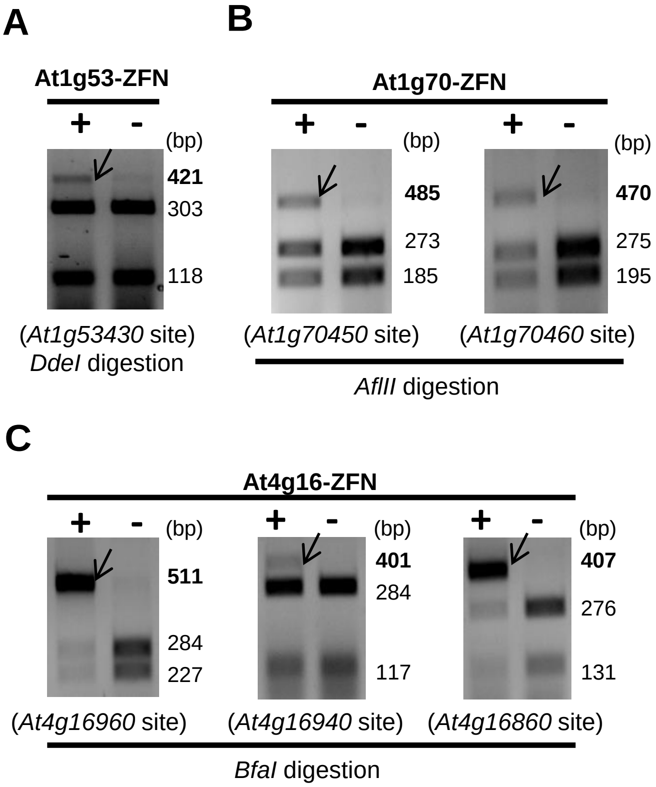

Figure S4 CoDA-assembled ZFNs are active in T1 plants. (A) At1g53-ZFN's activity is detected at *At1g53430*. (B) At1g70-ZFN's activity is detected at both *At1g70450* and *At1g70460*. (C) At4g16-ZFN's activity is detected at *At4g16960*, *At4g16940* and *At4g16860.* Activity of ZFNs was measured by enrichment PCR using the restriction enzymes shown in each panel. The uncut bands represent ZFN-induced mutations and are indicated by arrows. Bulked estradiol-treated T1 transgenic plants or wild type plants were compared.
