# Supplementary material for: Targeted Deletion and Inversion of Tandemly Arrayed Genes in Arabidopsis thaliana Using Zinc Finger Nucleases
Source: G3 (Bethesda). 2013 Oct 1;3(10):1707–15. doi: 10.1534/g3.113.006270 (PMC3789795; doi:10.1534/g3.113.006270)
Supplement: Supporting Information [file supp_g3.113.006270_FigureS6.pdf]

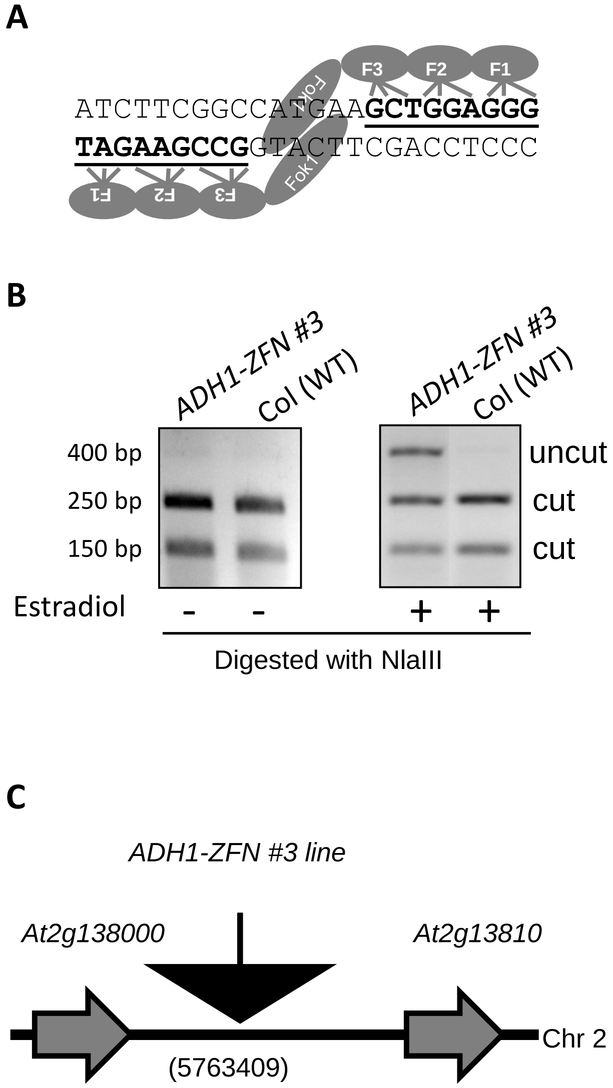

Figure S6 An active ADH1-ZFN #3 line. (A) Schematic of the ADH1-ZFN and its target site. (B) ADH1-ZFN activity is highly estradiol-inducible. Mutagenesis activity, as reflected by the uncut band, was detected by PCR and digestion (C) Precise location of the transgene in *ADH1-ZFN* #3 line as mapped by TAIL-PCR.
