# Supplementary material for: Targeted Deletion and Inversion of Tandemly Arrayed Genes in Arabidopsis thaliana Using Zinc Finger Nucleases
Source: G3 (Bethesda). 2013 Oct 1;3(10):1707–15. doi: 10.1534/g3.113.006270 (PMC3789795; doi:10.1534/g3.113.006270)
Supplement: Supporting Information [file supp_g3.113.006270_FigureS7.pdf]

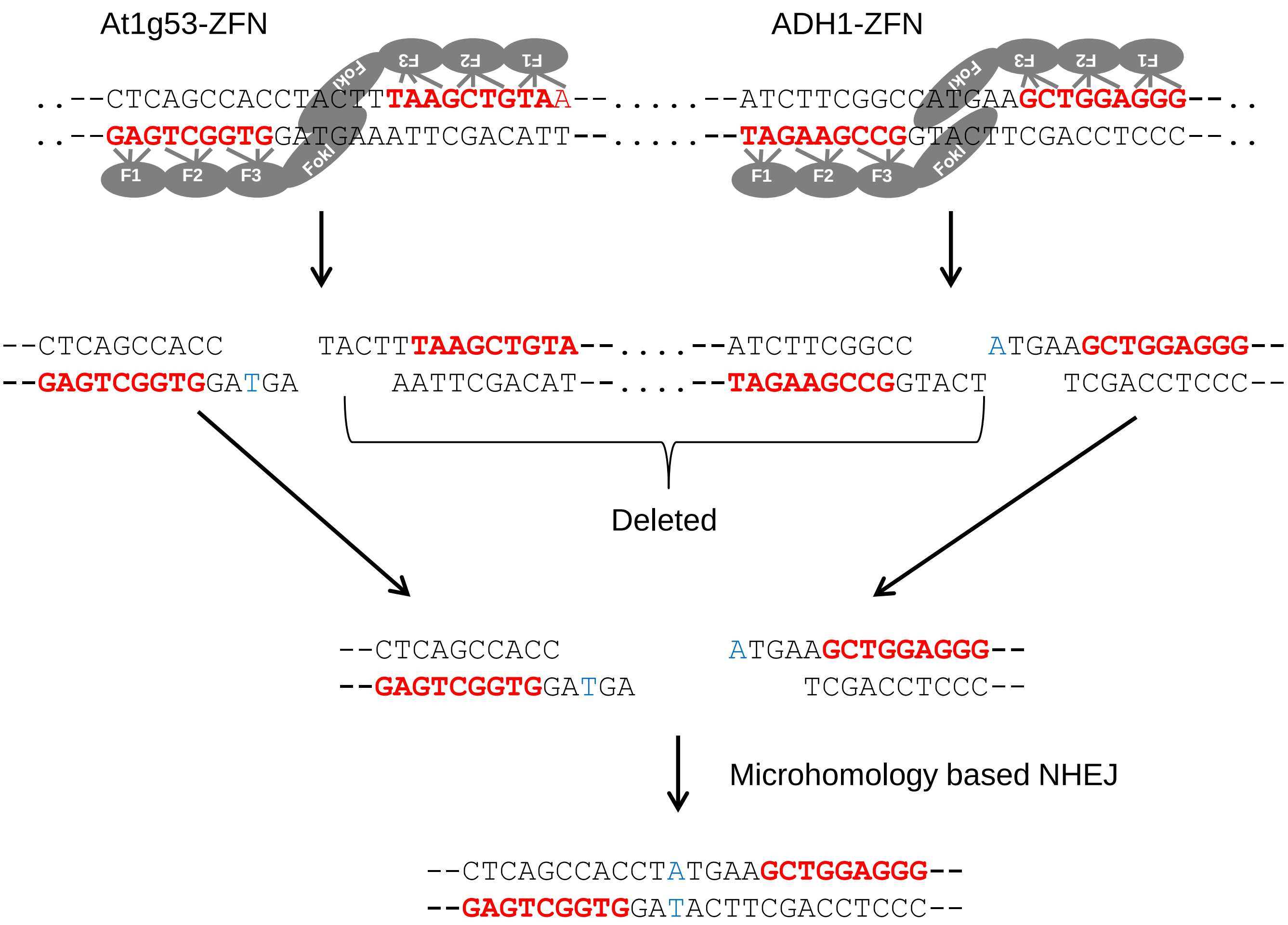

Figure S7 A possible NHEJ repair mechanism using 1-bp of microhomology. The process that leads to a common ligation product is depicted. The ZFN binding sites are shown in red and the 1 nt of likely microhomology is marked in blue.
