# Supplementary material for: Targeted Deletion and Inversion of Tandemly Arrayed Genes in Arabidopsis thaliana Using Zinc Finger Nucleases
Source: G3 (Bethesda). 2013 Oct 1;3(10):1707–15. doi: 10.1534/g3.113.006270 (PMC3789795; doi:10.1534/g3.113.006270)
Supplement: Supporting Information [file supp_g3.113.006270_FigureS8.pdf]

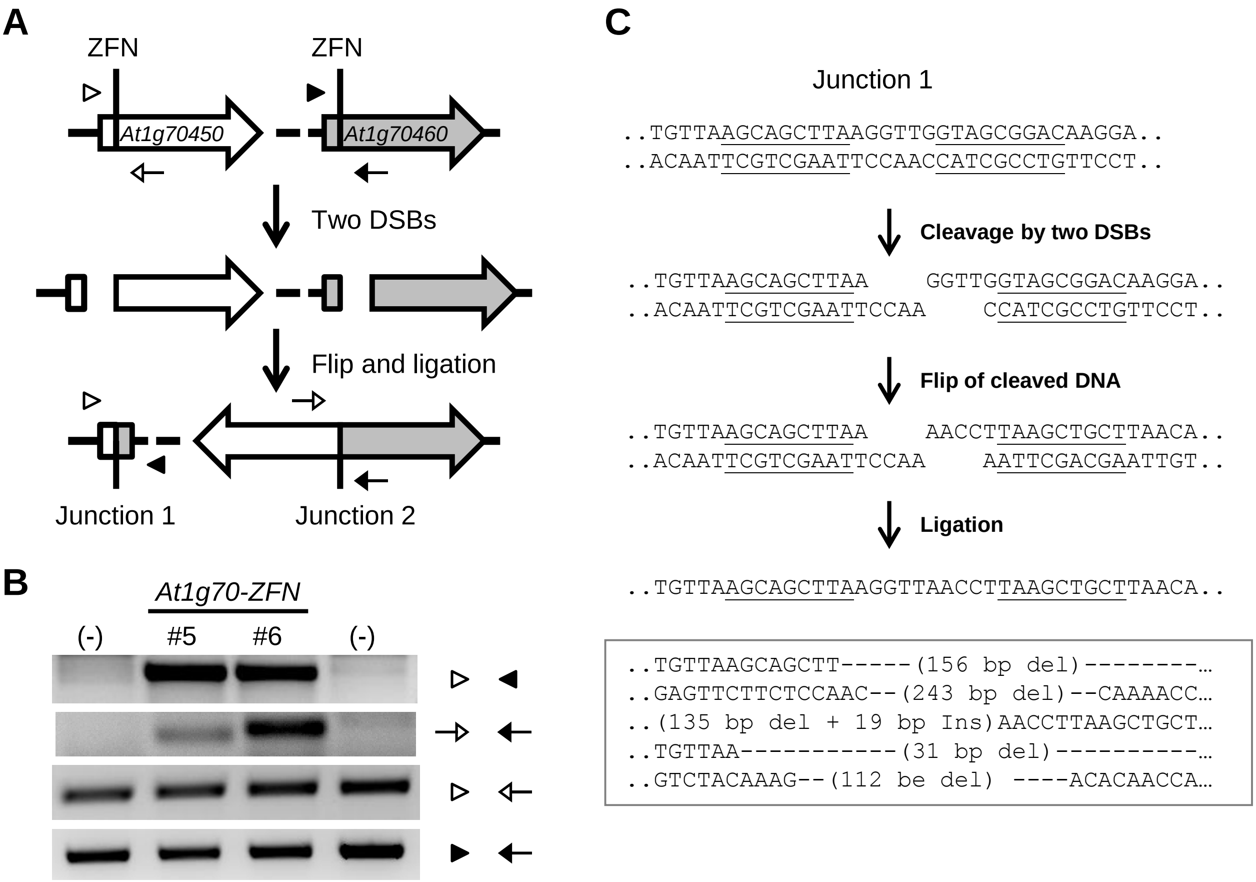

Figure S8 Inversion of the At1g70450 gene cluster. (A) Schematic of the *At1g70450* gene cluster inversion. Positions of PCR primers for confirming inversions are indicated by empty or filled triangles and arrows. (B) PCR confirmation of gene cluster inversions. (C) DNA sequence confirmation of inversions.
