# Supplementary material for: Targeted Deletion and Inversion of Tandemly Arrayed Genes in Arabidopsis thaliana Using Zinc Finger Nucleases
Source: G3 (Bethesda). 2013 Oct 1;3(10):1707–15. doi: 10.1534/g3.113.006270 (PMC3789795; doi:10.1534/g3.113.006270)
Supplement: Supporting Information [file supp_g3.113.006270_FigureS9.pdf]

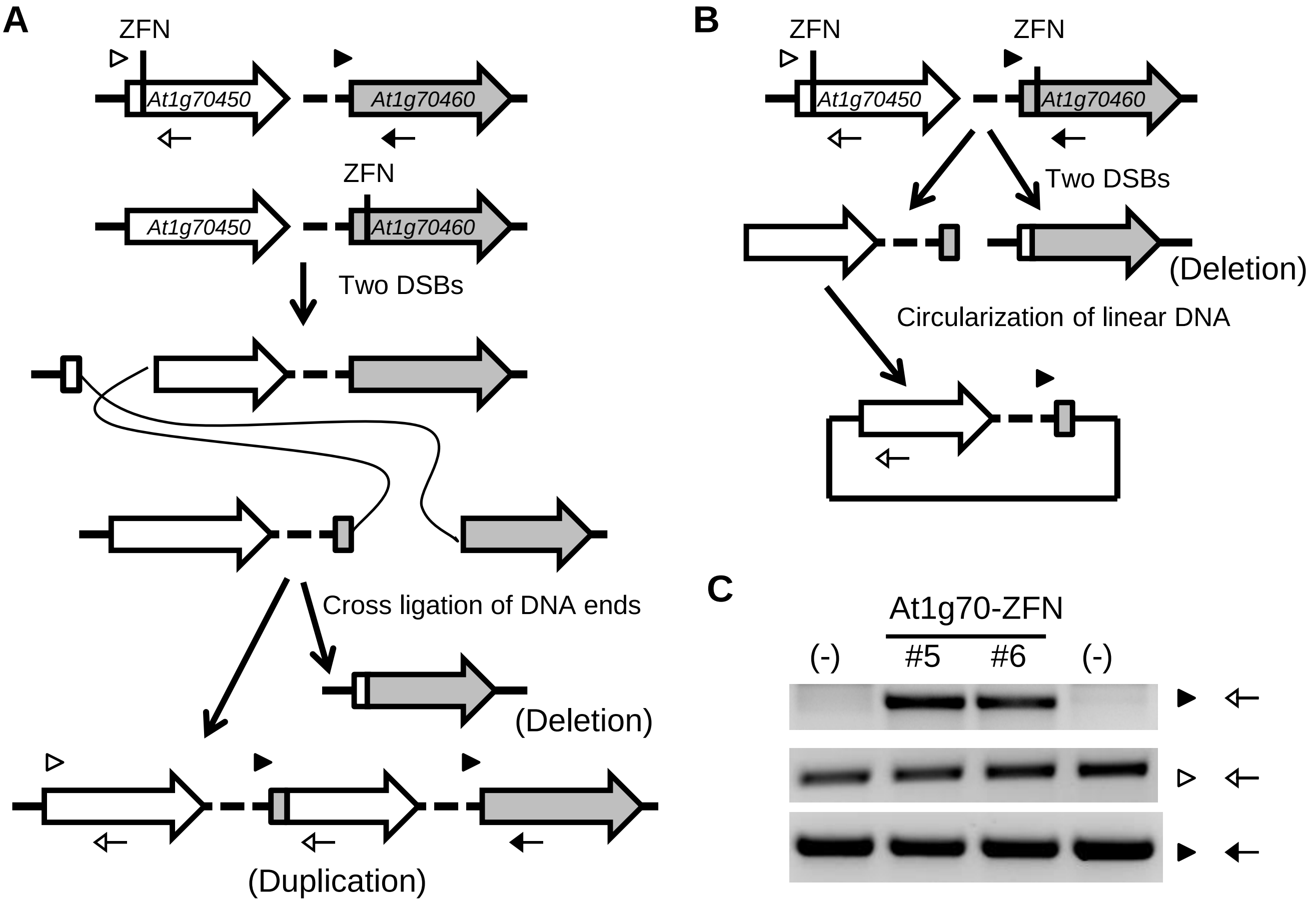

D

TGTTAAGCAGCTTAAGGTTGGTAGCGGACAAGGT (PL)

TGTTAAGCAGCTTAAGGTTGGTAGCGGACAAGGT (PL,20X)

TGTTAAGCAGCTTAAGGgTTGGTAGCGGACAAGG +1

TGTTAAGCAGCTTAAGGTTtGGTAGCGGACAAGG +1 (6X)

TGTTAAG-AGCTTAAGGTTGGTAGCGGACAAGGT -1

TGTTAAGCAGCTTAAGG---GTAGCGGACAAGGT -3

TGTTAAGCAGCTTAAGGTT---AGCGGACAAGGT -3

TGTTAAGCAGCTTAA---TGGTAGCGGACAAGGT -3

TGTTAAG--------------------------- -42

-----------------------GCGGACAAGGT -48

Figure S9 Duplication of a gene cluster or circularization of deleted DNA at the At1g70450-At1g70460 locus. (A) Schematic of the *At1g70450* gene cluster duplication. (B) Schematic of circularization of deleted DNA. (C) PCR confirmation of possible gene cluster duplications. (D) DNA sequence data from clones indicative of possible gene cluster duplications.
