# Supplementary material for: Targeted Deletion and Inversion of Tandemly Arrayed Genes in Arabidopsis thaliana Using Zinc Finger Nucleases
Source: G3 (Bethesda). 2013 Oct 1;3(10):1707–15. doi: 10.1534/g3.113.006270 (PMC3789795; doi:10.1534/g3.113.006270)
Supplement: Supporting Information [file supp_g3.113.006270_TableS1.pdf]

Table S1 Zinc finger arrays, recognition sites and recognition helices.

Zinc finger arrays	Recognition sites and recognition helix amino acid sequences		
	F1	F2	F3
At1g53-ZF_left	GAG	GCT	GTG
	KHSNLTR	QRSDLTR	RPDALPR
At1g53-ZF_right	GTA	GCT	TAA
	QQSSLLR	QRSDLTR	QRGNLNM
At1g70-ZF_left	GCT	GCT	TAA
	MKNTLTR	QRSDLTR	QRGNLNM
At1g70-ZF_right	GAC	GCG	GTA
	DPSNLIR	RTDTLAR	QGGALQR
At4g16-ZF_left	GAA	GAA	GAA
	QASNLTR	QQTNLTR	QTNNLNR
At4g16-ZF_right	GGA	GCC	GTA
	DNAHLAR	DSSVLRR	QSTSLQR
At3g21-ZF_left	TGT	GCT	GGT
	KRQHLEY	QRSDLTR	HGHRLKT
At3g21-ZF_right	GCT	GCC	GAT
	LRTSLVR	DSSVLRR	LSTNLTR
At5g01-ZF_left	GGA	GGC	GGA
	RPSKLVL	LKEHLTR	QSQHLVR
At5g01-ZF_right	GAA	GAA	GGC
	QASNLTR	QQTNLTR	KNVSLTH
