# Supplementary material for: Targeted Deletion and Inversion of Tandemly Arrayed Genes in Arabidopsis thaliana Using Zinc Finger Nucleases
Source: G3 (Bethesda). 2013 Oct 1;3(10):1707–15. doi: 10.1534/g3.113.006270 (PMC3789795; doi:10.1534/g3.113.006270)
Supplement: Supporting Information [file supp_g3.113.006270_TableS2.pdf]

Table S2 Oligos for amplifying Arabidopsis DNA

Oligo name	Oligo sequence	Purpose	Note
At1g53430-F2	CTgtaagcaaaactaactaaccac	For detection of mutations at At1g53430 site	
At1g53430-R2	ctcacGTTTAGCATCTTCTGGACA	For detection of mutations at At1g53430 site and gene cluster inversions	Designated as an open arrow in Fig. S5A and B
At1g53440-F1	tagatgatatttttaaccgtgac	For detection of gene cluster inversions and large chromosomal deletions	Designated as F2 in Fig. 4A and as a filled, tailess arrow in Fig. 5A and B
At1g53440-F2	tattcggtatcatcaaggtca	For detection of mutations at At1g53440 site	
At1g53440-R2	TTCTTAAGCACCATTGGACActac	For detection of mutations at At1g53440 site	
At1g53430-F1	ATTGGTTCCATGAGTGAGC	For detection of gene cluster deletions, inversions and large chromosomal deletions	Designated as F1 in Fig. 3A and Fig. 4A, and as an open tailess arrow in Fig. 5A and B
At1g53440-R3	aagggcttcttttttcaag	For detection of gene cluster deletions and inversions	Designated as R2 in Fig. 3A and as a filled arrow in Fig. 5A and B
At1g70450-F3	TTCTTCTCCAACAGCACCGTCAG	For detection of mutations at At1g70450 site and gene cluster deletions	Designated as F1 in Fig. 3B
At1g70450-R2	CACTGGCCTACCCTTCCctgtc	For detection of mutations at At1g70450 site and gene cluster inversions	Designated as an open arrow in Fig. S8A and B
At1g70450-R3	GGTGACctgcaaaacaagataaat	For detection of gene cluster duplications	Designated as an open arrow in Fig. S9
At1g70460-F	TACTCTGGTCCTGGTGGTTACAAT	For detection of gene cluster duplications	Designated as a tailess filled arrow in Fig. S9
At1g70460-F3	GAGGAGGAGGTTATACACGGTCAG	For detection of mutations at At1g70460 site	
At1g70460-R2	AGTACTGGCCTTCCCTTCCctatc	For detection of mutations at At1g70460 site and gene cluster inversions	Designated as an filled arrow in Fig. S8A and B
At1g70460-R3	tgcaaaacaaaacaaaaacataca	For detection of gene cluster deletions	Designated as R2 in Fig. 3B
At4g16960-F	gtcttgttaggtggtttgatgtta	For detection of NHEJ-mediated mutagenesis	
At4g16960/940-R	CCATTTGATCCAAGTCTTTG	For detection of mutations at At4g16960 and At4g16940 sites	
At4g16940-F	agcaccacctcagccccatac	For detection of mutations at At4g16960 site and gene cluster deletions	Designated as F2 in Fig. 3C
At4g16860/950-F	tggagggaaggaagacgaagtt	For detection of mutations at At4g16860 site	
At4g16860-R	ATTTGTTCCCCTTTCTTGTA	For detection of mutations at At4g16860 site and gene cluster deletions	
At4g16960-F2	tctgtatcatattagtttagttcg	For detection of gene cluster deletions	Designated as F1 in Fig. 3C
At4g16940-R2	aaagagaataacacagatttattt	For detection of gene cluster deletions	Designated as R2 in Fig. 3C
ADH1F	TCGAGGAAGTGGAGGTTGCT	For detection of mutations at the ADH1 site	
ADH1R2	TGGCTGAAGATCAGTCACTCC	For detection of mutations at the ADH1 site and large chromosomal deletions	Designated as R in Fig. 4A
