# Supplementary material for: Targeted Deletion and Inversion of Tandemly Arrayed Genes in Arabidopsis thaliana Using Zinc Finger Nucleases
Source: G3 (Bethesda). 2013 Oct 1;3(10):1707–15. doi: 10.1534/g3.113.006270 (PMC3789795; doi:10.1534/g3.113.006270)
Supplement: Supporting Information [file supp_g3.113.006270_FigureS2.pdf]

A

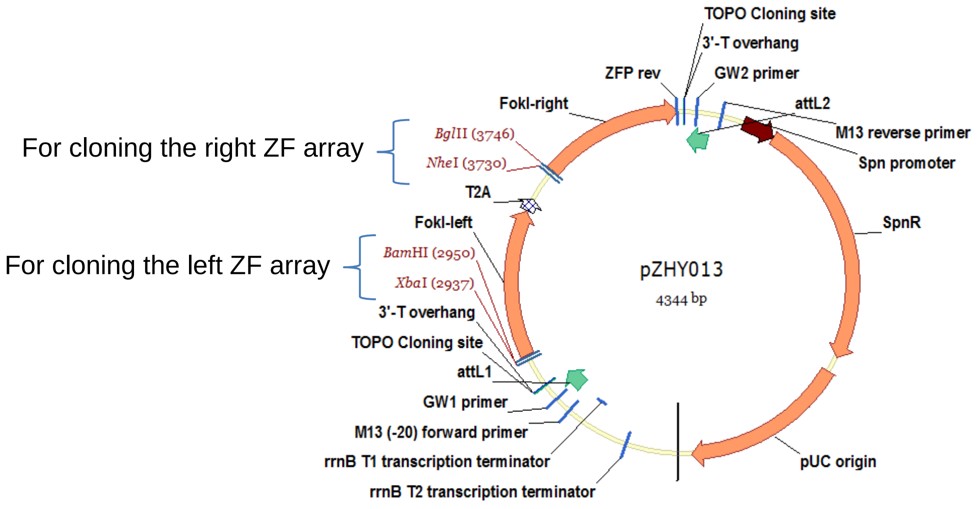

B

>pZHY013
aagggcgaattcgacccagctttcttgtacaaagttggcattataaaaaataattgctcatcaatttgttgcaacgaacaggtcactatcagtcaaaataaaatcattatttgccatccagctga tatcccctatagtgagtcgtattacatggtc
atagctgtttcctggcagctctggcccgtgtctcaaaatctctgatgttacattgcacaagataaaaatatatcatcatgcctcctctggaccagccaggacagaaatgcctcgacttcgctgctgcccaaggttgccgggtgacgcacaccg
tggaaacggatgaaggcacgaacccagtggacataagcctgttcggttcgtaagctgtaatgcaagtagcgtatgcgctcacgcaactggtccagaaccttgaccgaacgcagcggtggtaacgg cgcagtggcggttttcatggcttg
ttatgactgtttttttggggtacagtctatgcctcgggcatccaagcagcaagcgcgttacgccgtgggtcgatgtttgatgttatggagcagcaacgatgttacgcagcagggcagtcgcccta aaacaaagttaaacatcatgagggaag
cggtgatcgccgaagtatcgactcaactatcagaggtagttggcgtcatcgagcgccatctcgaaccgacgttgctggccgtacatttgtacggctccgcagtggatggcggcctgaagccacac agtgatattgatttgctggttacggtg
accgtaaggcttgatgaaacaacgcggcgagctttgatcaacgaccttttggaaacttcggcttcccctggagagagcgagattctccgcgctgtagaagtcaccattgttgtgcacgacgacat cattccgtggcgttatccagctaagcg
cgaactgcaatttggagaatggcagcgcaatgacattcttgcaggtatcttcgagccagccacgatcgacattgatctggctatcttgctgacaaaagcaagagaacatagcgttgccttggtag gtccagcggcggaggaactctttgat
ccggttcctgaacaggatctatttgaggcgctaaatgaaaccttaacgctatggaactcgccgcccgactgggctggcgatgagcgaaatgtagtgcttacgttgtcccgcatttggtacagcgc agtaaccggcaaaatcgcgccgaa
ggatgtcgctgccgactgggcaatggagcgcctgccggcccagtatcagcccgtcatacttgaagctagacaggcttatcttggacaagaagaagatcgcttggcctcgcgcgcagatcagttgg aagaatttgtccactacgtgaaag
gcgagatcaccaaggtagtcggcaaataaccctcgagccacccatgaccaaaatcccttaacgtgagttacgcgtcgttccactgagcgtcagaccccgtagaaaagatcaaaggatcttcttga gatcctttttttctgcgcgtaatctgct
gcttgcaaacaaaaaaaccaccgctaccagcggtggtttgtttgccggatcaagagctaccaactctttttccgaaggtaactggcttcagcagagcgcagataccaaatactgtccttctagtg tagccgtagttaggccaccacttcaag
aactctgtagcaccgcctacatacctcgctctgctaatcctgttaccagtggctgctgccagtggcgataagtcgtgtcttaccgggttggactcaagacgatagttaccggataaggcgcagcg gtcgggctgaacggggggttcgtgca
cacagcccagcttggagcgaacgacctacaccgaactgagatacctacagcgtgagcattgagaaagcgccacgcttcccgaagggagaaaggcggacaggtatccggtaagcggcagggtcgga acaggagagcgcacga
gggagcttccagggggaaacgcctggtatctttatagtcctgtcgggtttcgccacctctgacttgagcgtcgatttttgtgatgctcgtcaggggggcggagcctatggaaaaacgccagcaac gcggcctttttacggttcctggccttttgct
ggccttttgctcacatgttctttcctgcgttatcccctgattctgtggataaccgtattaccgcctttgagtgagctgataccgctcgccgcagccgaacgaccgagcgcagcgagtcagtgagc gaggaagcggaagagcgcccaatacg
caaaccgcctctccccgcgcgttggccgattcattaatgcagctggcacgacaggtttcccgactggaaagcgggcagtgagcgcaacgcaattaatacgcgtaccgctcgccaggaagagtttg tagaaacgcaaaaaggccatc
cgtcaggatggccttctgcttagtttgatgcctggcagtttatggcgggcgtcctgcccgccaccctccgggccgttgcttcacaacgttcaaatccgctcccggcggatttgtcctactcagga gagcgttcaccgacaaacaacagataaa
acgaaaggcccagtcttccgactgagcctttcgttttatttgatgcctggcagttccctactctcgcgttaacgcttgcatggatgttttcccagtcacgacgttgtaaaacgacggccagtctt aagctcgggccccaaataatgattttattttgac
tgatagtgacctgttcgttgcaacaaattgatgagcaatgcttttttataatgccaactttgtacaaaaaagcaggctccgaattcgcccttcaccatggattataaggatcacgatggcgacta caaggaccacgatattgactacaaagac
gatgacgataaaatggctcctaagaaaaagcgcaaagtcggtatccatggcgttccctctagataacgcaggatcccagctggtgaagtccgagctggaagaaaaaaagagcgagctgcgccaca agctcaagtacgtgccccac
gagtacatcgagctgatcgagatcgcccgcaacagcacccaagaccgcatcctggagatgaaagtgatggagttcttcatgaaggtgtacggctaccgcggcaagcacctgggcggctcccgcaa gcccgatggcgccatctaca
ccgtgggctcccccatcgactatggcgtcattgtcgacaccaaggcctactccggcggctacaacttacccatcggtcaggccgacgagatgcaacgctacgtgaaggagaaccagacccgcaat aagcacattaatcccaacgag
tggtggaaggtgtacccctcctccgtgaccgagttcaaattcctgttcgtgtccggccacttcaagggcaattataaggcccaactgacccgcctgaaccacaagaccaactgcaacggcgccgt gctgtccgtggaggaactgctgatc
ggcggcgagatgatcaaggctggtaccctgaccctggaagaggtgcgccgcaagttcaacaatggtgaaatcaatttcaggtccggcggcggagagggcagaggaagtcttctaacatgcggtga cgtggaggagaatcccggcc
ctaggatggactacaaagaccatgacggtgattataaagatcatgacatcgattacaaggatgacgatgacaagatggcccccaagaagaagaggaaggtgggcattcacggggtgccggctagc tagtaagcttagatctcaact
agtcaaaagtgaactggaggagaagaaatctgaacttcgtcataaattgaaatatgtgcctcatgaatatattgaattaattgaaattgccagaaattccactcaggatagaattcttgaaatga aggtaatggaatttttatgaaagtttatg
gatatagaggtaaacatttgggtggatcaaggaaaccggacggagcaatttatactgtcggatctcctattgattacggtgtgatcgtggatactaaagcttatagcggaggttataatctgcca attggccaagcagatgaaatggagcg
atatgtcgaagaaaatcaaacacgaaacaaacatctcaaccctaatgaatggtggaaagtctatccatcttctgtaacggaatttaagtttttatttgtgagtggtcactttaaaggaaactaca aagctcagcttacacgattaaatcatatc
actaattgtaatggagctgttcttagtgtagaagagcttttaattggtggagaaatgattaaagccggcacattaaccttagaggaagtgagacggaaatttaataacggcgagataaactttta atag

Figure S2 ZFN expression entry clone-pZHY013. (A) Map of the pZHY013 entry clone. The restriction enzyme sites for cloning zinc finger arrays are marked. (B) Full sequence of the pZHY013 entry clone.
