# Supplementary material for: Targeted Deletion and Inversion of Tandemly Arrayed Genes in Arabidopsis thaliana Using Zinc Finger Nucleases
Source: G3 (Bethesda). 2013 Oct 1;3(10):1707–15. doi: 10.1534/g3.113.006270 (PMC3789795; doi:10.1534/g3.113.006270)
Supplement: Supporting Information [file supp_g3.113.006270_FigureS5.pdf]

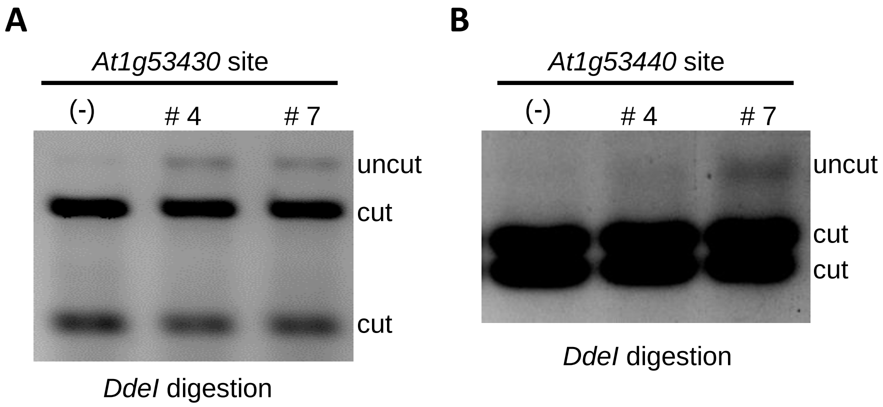

Figure S5 At1g53-ZFN activity at two targets revealed by enrichment PCR in T2 plants. (A) Enrichment PCR detection of ZFN activity at the *At1g53430* locus in two T2 transgenic populations. (B) Enrichment PCR detection of ZFN activity at the *At1g53440* locus in two T2 transgenic plant populations.
